# Prevalence of common symptoms of neonatal illness in Northwest Ethiopia: A repeated measure cross-sectional study

**DOI:** 10.1371/journal.pone.0248678

**Published:** 2021-03-30

**Authors:** Tadesse Guadu Delele, Gashaw Andargie Biks, Solomon Mekonnen Abebe, Zemene Tigabu Kebede

**Affiliations:** 1 Department of Environmental and Occupational Health, and Safety, Institute of Public Health, College of Medicine and Health Sciences, University of Gondar, Gondar, Ethiopia; 2 Departments of Health System and Policy, Institute of Public Health, College of Medicine and Health Sciences, University of Gondar, Gondar, Ethiopia; 3 Departments of Human Nutrition, Institute of Public Health, College of Medicine and Health Sciences, University of Gondar, Gondar, Ethiopia; 4 Departments of Pediatrics and Child Health, School of Medicine, College of Medicine and Health Sciences, University of Gondar, Gondar, Ethiopia; University of Mississippi Medical Center, UNITED STATES

## Abstract

**Background:**

The neonatal period is the most vulnerable stage of life. In Ethiopia, neonatal illness is common and the reduction in neonatal mortality is not as significant as for under-five mortality.

**Objectives:**

To determine the prevalence and factors associated with neonatal illness symptoms reported by mothers delivering in health facilities in Northwest Ethiopia.

**Methods:**

A repeated measure cross-sectional study design was employed to collect data from 358 randomly selected deliveries in 11 health facilities from November 2018 to March 2019. A pretested and interviewer-administered structured questionnaire adapted from the literature was employed to record neonatal outcomes (illnesses and/or deaths) at birth, 24 hours, 7^th^, 14^th^ and 28^th^ day from birth. Cleaned data was exported to STATA version 14 software for analysis. Multilevel analysis was used to identify individual and facility-level characteristics associated with neonatal illness symptoms.

**Results:**

The prevalence of neonatal illness symptoms was 27.8% (95% CI; 23.2, 32.8) of the 338 babies born alive and the neonatal mortality rate was 41/1000 live births (14/338). The most common symptoms or conditions of neonatal illness reported by mothers’ in the study area were possible serious bacterial infections (95.8%, 90/94), localized bacterial infections (43.6%, 41/94), low birth weight (23.4%, 22/94), diarrhea (18.1%, 17/94), prematurity (14.9%, 14/94), and jaundice (7.5%, 7/94). Among the babies who died, neonates who had possible serious bacterial infections, low birth weight, localized bacterial infections, and prematurity took the highest proportions with 100% (14/14), 64.3% (9/14), 50% (7/14), and 42.9% (6/14), respectively. Having a maximum of 3 children (AOR = 1.96; 95% CI = 1.1–3.6), having twins or triplets during pregnancy (AOR = 2.43; 95% CI = 1.1–6.1), and lack of antenatal counseling (AOR = 1.83; 95% CI = 1.1–3.3) were among the maternal factors associated with neonatal illness. Having low birth length (AOR = 7.93; 95% CI = 3.6–17.3), and having a poor breastfeeding quality (AOR = 2.37; 95% CI = 1.4–4.0) were found to be the neonatal factors associated with neonatal illness.

**Conclusions:**

This study indicated a high prevalence of neonatal illness symptoms in Northwest Ethiopia. Therefore, early detection, referral and better management of symptoms or conditions with a high mortality, like sepsis and low birth weight are compulsory to save the lives of many neonates. Strengthening the health extension programme to improve antenatal care service utilization and breastfeeding quality of neonates among postpartum women is crucial.

## Background

The global number of neonatal deaths declined from 5 million in 1990 to 2.5 million in 2018. On the other hand, neonatal mortality accounted for about 40% of all under-five deaths in 1990, while its proportion increased to 47% in 2018 [1]. Africa contributed to one-third of the world’s neonatal mortality burden. Among (Sustainable Developmental Goals (SDG) regions, Sub-Saharan Africa had the highest neonatal mortality rate in 2017 and 2019 at 27 deaths per 1000 live births [2,3–5].

The major illness conditions which caused the highest proportion of neonatal deaths in 2012 globally were reported to be infections (35%), preterm births (28%), intrapartum related complication (24%), and asphyxia (23%) [6]. The most common illnesses leading to neonatal mortality in Ethiopia were infection, asphyxia, and preterm birth in 2019 [7].

In Ethiopia changes in neonatal mortality are not as substantial as changes in under-five mortality. The rate of under-five mortality (U5MR) had decreased by 55% from the year 2005 through 2019; i.e. from 123 to 55 per 1000 of live births. However, the neonatal mortality rate had decreased only from 39 to 30 per 1000 live births from 2005 to 2019. Because of this, the share of neonatal mortality in under-five mortality has increased from 31% to 55% [8].

Furthermore, many women do not generally seek formal healthcare during pregnancy, childbirth, and puerperium in Northwest Ethiopia. Less than a third of women receive antenatal care and 90% are assisted by unskilled attendants: TBAs (26%) relatives (58%) or alone (6%). Almost no one (3.5%) receives postnatal care [9].

Neonatal illness and mortality is a reflection of the socioeconomic status of a society [10]. Improved neonatal survival requires not only to care before, during and after pregnancy; but also, wider issues of socioeconomic development including a reduction in poverty and increased maternal education [8–10]. Similarly, strategies to reduce neonatal mortality should cover the whole midwife-led continuum of care from maternal health before and during pregnancy to delivery, and early neonatal care to child health programs [9].

Different tools have been introduced to the health programs of many countries including Ethiopia to facilitate early identification and management of neonatal illnesses. One of these programs is the Integrated Management of Childhood Illness (IMCI) developed by WHO, one of the focuses being detection of neonatal illnesses and providing prompt timely treatment [11]. Despite the availability of this lifesaving document, health facilities, and care providers in Ethiopia rarely used to report neonatal illnesses [12].

Newborn survival is an issue of great concern to developing countries, like Ethiopia. Care for ill neonates often receives little attention in maternal and child health programs. Despite the various efforts that have been made by the Ethiopian government to reduce neonatal mortality recent studies showed that it is still high [9,10,13]. There are no studies about newborn illness reported by mothers, and/or mother-and baby pair during postnatal care, prevalence of neonatal illness is not known. Furthermore, due to the fact that women do not seek health care, only very few localized studies on sepsis have reported it’s prevalence [14,15] among neonates admitted to neonatal intensive care unit in Northwest Ethiopia. Therefore, this research aimed to determine the prevalence and determinant factors of neonatal illness symptoms commonly reported by mothers’ in Northwest Ethiopia.

## Methods

### Ethical considerations

Before the start of data collection, ethical clearance was obtained from the Institutional Review Board of the University of Gondar (UoG) and permission letters were secured from officials of districts and health institutions. During data collection, the study participants were asked to participate voluntarily, communicated privately and signed written agreements on consent forms. They were ensured that their responses would be kept confidentially.

### Study design and health facility setting

A repeated measure cross-sectional study design was used to measure the prevalence of neonatal illnesses commonly perceived by facility delivered mothers in North Gondar Zone from November 2018 through March 2019. The zone is structured in eight districts, including two town administrations. This administrative zone had an estimated total population of 876,255. Currently, two district hospitals and 35 health centers are providing delivery services in this administrative area using 1116 healthcare workers [12].

**Fig 1:**
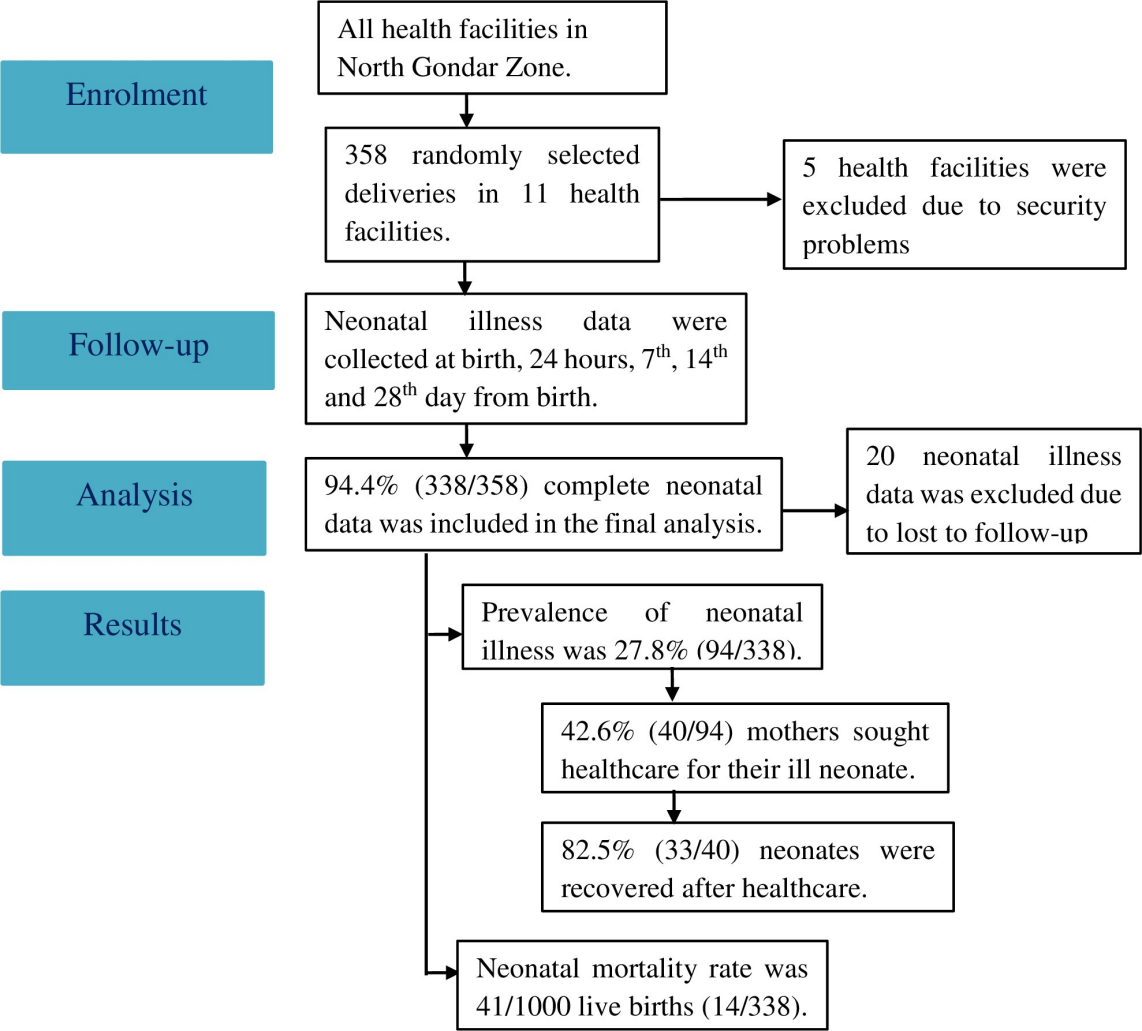
Study flow diagram of neonatal illness follow-up data collected among health facility deliveries in Northwest Ethiopia, March 2019.

Three districts (Dabat Zuria, Debark, and Janamora) were randomly selected. All facilities providing delivery services (two hospitals and fourteen health centers) in the randomly selected districts were included in the study. However, five health centers were excluded due to lack of deliveries during data collection period owing to security problems. Hence, eleven health facilities were eventually used to collect the data on delivery and service provision (Fig 1). In terms of healthcare, all the basic essential newborn care services are available during delivery in both district hospitals and health centers. Nevertheless, a higher level of newborn care is expected in district hospitals due to the availability of better neonatal intensive care equipment and specialists. Health centers usually use referral system to their nearby district hospital for cases that need a better level of newborn care.

### Source and study population

All neonates in North Gondar Zone delivered in governmental health facilities were the source population. Those neonates delivered in randomly selected district health facilities were the study populations. All neonates delivered in the randomly selected district health facilities and randomly selected for inclusion during the data collection period were the study subjects.

### Inclusion and exclusion criteria

The inclusion criteria for this study were being born alive in one of the study sites within the study period, and ability of the mother to provide consent. A sample from the eligible newborns was randomly selected and mothers were asked for consent to participate. In case of twins or triplets, which share maternal, health facility and newborn care quality related characteristics in common, one of them was selected using lottery method to avoid selection bias.

### Variables and operational definitions

#### Dependent variable

The occurrence of neonatal illness symptoms perceived by mothers.

#### Independent variables

Socio-demographic, obstetric, facility and newborn care quality-related characteristics were the independent variables.

**Neonatal illness** is the occurrence of at least one perceived symptom of neonatal illness during the neonatal period based on the systematic inflammatory response syndrome (SIRS) definition [13]. Neonates who showed at least one of the symptoms, as reported by their mother at least once during the neonatal period were labeled as “YES”, and “NO” if they did not face any of them. The groups of illness symptoms or conditions derived from the adapted SIRS’s definition set by the integrated management of childhood illness (IMCI) includes; possible serious bacterial infection (PSBI), localized bacterial infection (LBI), Low birth weight (LBW), prematurity, diarrhea, and jaundice (S1 Annex).

**Prematurity** is defined as any newborn delivered before 37 weeks of gestation from last menstrual period reported by mothers.

**Neonatal mortality rate** is defined as the number of neonatal deaths per 1000 live births.

**Essential newborn care** is a set of practices provided by healthcare workers and mothers to every newborn during delivery. **Cord care** is one component of essential newborn care, which refers to the use of a clean cutting instrument to cut the umbilical cord (boiled new, used blade or scissor) plus clean thread, cord tie or cord clamp and no any substance applied on the cord stump. Similarly, **breastfeeding** is another component of essential newborn care, which refers to the initiation of breastfeeding within the first one hour after birth, giving no prelacteal and feeding the child with colostrum. Cord care and breastfeeding practices were assessed using YES/NO question items. Then, a composite variable from these questions was generated to categorize each component as having “Good/Poor quality”. The component was categorized as good quality if it scored mean and above the mean value, and poor quality if otherwise.

**Essential newborn care quality** was assessed using a validated thirty-two question items questionnaire and the quality score was categorized as good if they scored mean and above the mean value of the items correctly, and poor quality if otherwise. The detail of the measurement and tool was found in the recent publication [16].

### Sample size and sampling technique

The total sample of the study was determined by using a single population proportion formula by assuming a 3% level of significance, 5% margin error and taking 77.8% proportion of neonatal illness due to sepsis and 88.6% for ANC attendance for factors from a study done at the University of Gondar Specialized Hospital [15]. Considering a 10% non-response rate, the final sample size was 358 newborns.

$$N=\frac{{\left(\mathrm{Z\alpha}/2\right)}^{2}x\;p\;\left(1-p\right)}{d^{2}}$$

A cluster sampling technique was employed to select the neonates, who were born alive. First, districts were randomly selected and then, all health facilities in those randomly selected districts were included. A systematic random sampling technique was employed to select the mothers, who deliver a live birth/s. The sample size was proportionally allocated to health centers and hospitals based on the respective health facilities prior annual ANC attendance. Data was collected from eligible newborns.

### Data collection tool and method

A pretested, interviewer-administered structured questionnaire adapted from different kinds of literature [9–13,16] were employed to collect data. The questionnaire was designed to record information regarding the mothers’ socio-demographic characteristics, obstetric and neonatal illness histories during the neonatal period. The IMCI and SIRS definitions of neonatal illness were used to collect illness symptoms or conditions after adaption in local contexts. BEmONC and Child immunization services readiness levels were also assessed by adopting WHO BEmONC and Child immunization service questionnaire domain items (Trained staff and guidelines, Equipment, Medicines and commodities). The pretest was done among 30 health facility delivered mothers outside the study area, but with similar characteristics. After pretest, the flow of the questionnaire and language of some words were revised. Five consecutive neonatal illness histories were collected by interviewing the mothers’ based on their perception and observation of symptoms. The first two neonatal illness histories together with the neonatal and maternal related data were collected in health facility at birth (after being stable), and 24 hours from birth, while the later three neonatal illness histories (7^th^, 14^th^ and 28^th^ day from birth) were collected through follow-up at home after a health facility delivery. Face to face interview was conducted by six trained data collectors who are first-degree female midwives and nurses. The consistency and completeness of the follow-up data were regularly checked by supervisors (clinicians and researchers) throughout the entire data collection period.

The questionnaire at the beginning was prepared in English (S2 Annex). The questionnaire was also translated into the local language, Amharic (S3 Annex), and back to English by translators who are bilingual and competent in the two languages to check the content validity.

### Data processing and analysis

The data was entered and cleaned using the Epi-Info version 7.1.5.0 software. Cleaning was done by running frequencies, proportions and summary statistics. The Principal Component Analysis (PCA) was employed to generate a wealth index [17]. The cleaned data was exported to STATA version 14 software for analysis.

### Descriptive analysis

Descriptive statistics were done to determine the prevalence of neonatal illness symptoms or conditions, and neonatal mortality. The proportion of the illness symptoms or conditions were presented against the total death outcomes. The prevalence of neonatal illness was calculated by dividing the total number of neonates who face illness symptoms or conditions at least once during the neonatal period from the total number of neonates who completed the follow-up period in percentage.

### Multilevel models

In this paper, the predictors of neonatal illness were assessed using a multilevel binary logistic regression. The choice of multilevel modeling was based on the following assumptions. First, the patterns of neonatal illness were influenced by the characteristics of different levels (individual, and health facility). Analyzing variables from different levels at one single common level using the standard binary logistic regression model leads to bias (loss of power or Type I error). Multilevel models allow us to consider the individual level and the facility level in the same analysis, rather than having to choose one or the other. Secondly, due to the repeated nature of the outcome variable (neonatal illness), neonates are likely to have repeated illnesses in repeated measurements. This fails to satisfy the assumption of independence among study subjects. Hence, the multilevel analysis is the appropriate method for such cases [18].

For the outcome variable (neonatal illness), four models were estimated. In model I (empty model) no determinant variables were included. This model represented the total variance of neonatal illness between individual and facility levels. In model II, individual (maternal and neonatal) level determinant variables were included. Model III, facility-level determinants were fitted. Model IV included all individual and facility-level variables which were significant at model II and III.

### Model building

A crude multilevel regression model was developed for each factor against the dependent variable. Bivariate logistic regression analysis was employed to identify the candidate explanatory variables at p-value<0.25 [19]. All models were built by using a stepwise forward method of model building technique. This method was chosen because of the shorter computation time it takes while running the models [20]. In addition to this, it is useful for developing the best prediction equation from the smallest number of variables.

### Parameter estimation methods

The measures of association (fixed-effects) estimate the association between the likelihood of neonatal illness and the predictor variables expressed as Adjusted Odds Ratio (AOR) with their 95% Confidence Intervals (CIs). The measures of variation (random-effects) were reported as the intra-class correlation coefficient (ICC) which is the percentage of variance explained by the community level variables.

### Comparison of models

Akaike Information Criterion (AIC) and log likelihood were used to compare the models [19]. The AIC and log likelihood values for each subsequent models were compared and the model with the lowest value was considered to be the better model.

### Multi-collinearity and model fit statistics

The presence of multicollinearity was checked among independent variables using the Variance Inflation Factor (VIF) at a cut-off point of 10. Predictors having a VIF value of less than 10 indicate the absence of multi-collinearity. In addition, Hosmer-Lemeshow goodness of fit test was used to estimate the goodness of fit of the adjusted final model [19].

## Results

### Health facility characteristics

The majority of the delivery sites were health centers; 9 (81.8%). The health facilities had 63.6% BEmONC service readiness score and 90.9% child immunization service readiness score levels. BEmONC trained staff and guidelines readiness score level was 27.2% (Table 1).

**Table 1:** Characteristics of health facilities in selected districts of North Gondar zone, Northwest Ethiopia, 2019.

Variable	Category	n	%
Administrative district	Dabat	04	36.4
	Debark	03	27.2
	Janamora	04	36.4
Delivery place	Health centers	09	81.8
	Hospitals	02	18.2
BEmONC trained staff and guidelines readiness score		03	27.2
BEmONC equipment readiness score		08	72.7
BEmONC medicines and commodities readiness score		10	90.9
BEmONC service readiness score		07	63.6
Child immunization service readiness score		10	90.9

### Maternal characteristics

A total of 338 mothers and 338 newborns (94.4%) were included in the follow up study, while the remaining 20 mothers were excluded in the final analysis due to loss in follow up. The mean age of the mothers was 26.4 years (SD = 5.6) with a minimum and maximum of 12 and 45 years, respectively. The majority of mothers were married; 325 (96.2%), and 253 (74.9%) were housewives (Table 2).

**Table 2:** Characteristics of mothers in selected districts of North Gondar zone, Northwest Ethiopia, 2019.

Variable	Category	n	%
Age group (years)	≤19	31	9.2
	20–29	202	59.8
	30–39	100	29.6
	≥ 40	05	1.5
Marital status	Married	325	96.2
	Not married*	13	3.8
Mothers education level	No education	124	36.7
	Primary	76	22.5
	Secondary	94	27.8
	More than secondary	44	13.0
Mothers occupation	Housewife	253	74.9
	Merchant	24	7.1
	Government employee	46	13.6
	Others**	15	4.4
Family size (number)	1–3	126	37.3
	≥ 4	212	62.7
Residence	Urban	174	51.5
	Rural	164	48.5
Household income	Poor	113	33.4
	Medium	112	33.2
	Rich	113	34.4
Source of household income	Agriculture	185	54.7
	Monthly salary	77	22.8
	Trade	45	13.3
	Others++	31	9.2
Health insurance usage	No	333	98.5
	Yes	05	1.5

* = Single, divorced, separated and living together ** = Daily laborer, private employ and student.

+ = Daily laborer and student ++ = Family support and daily labor.

### Obstetric characteristics of mothers

Most mothers 80.5% (272) had their first pregnancy after 18 years of age, 19.5% (48) deliveries were preterm, 69.8% (236) mothers had a history of three or less pregnancies, and 84.3% (285) mothers had antenatal care during the current pregnancy. Similarly, the majority 86.7% (293) of pregnancies were planned, 95.6% (323) of the pregnancies were singleton, 84.3% (285) deliveries were through spontaneous vaginal delivery (SVD) and 87.3% (295) deliveries were assisted by midwives (S4 Annex).

### Characteristics of newborns

Among the 338 newborns who completed the follow-up study, most 242 (72%) newborns were not visited by HEW after delivery, and 226 (67%) of them had no postnatal care visits to health facilities. Most neonates, 250 (73.9%) reached the nearest health center within an hour using the available transport, and 177 (52.4%) of them lived within two kilometers distance from the nearest health post (Table 3).

**Table 3:** Characteristics of newborns delivered in selected health facilities of North Gondar zone, Northwest Ethiopia (N = 338), 2019.

Variable	Category	N (%)	%
Birth weight (gram)	LBW/<2.5kg/	24	7.1
	NBW/2.5-4kg/	309	91.4
	Over/≥4kg/	05	1.5
Birth length (cm)	25–35	17	5
	36–55	321	95
Sex	Male	181	53.6
	Female	157	46.4
Stay at a health facility after delivery	< 24 hours	221	65.4
	≥ 24 hours	82	24.3
	I do not know	35	10.3
Postnatal care for neonates	No	226	66.9
	Yes	112	33.1
Visited by HEW after delivery	No	242	71.6
	Yes	96	28.4
Type of road to go to the nearest health center	No road at all	45	13.3
	Accessible in all weather conditions	197	58.3
	Accessible only in the winter season	35	10.4
	Asphalt road	61	18
Home distance from the nearest health center (hours)	Within an hour	250	73.9
	2–4 hours	79	23.4
	≥ 4 hours	09	2.7
Home distance from the nearest health post (km)	≤ 2	177	52.4
	2.1–5	106	31.4
	5.1–35	55	16.2

### Prevalence of symptoms

The prevalence of neonatal illness symptoms reported by the mother’s was 27.8% of all babies born alive, and neonatal mortality rate was 41/1000 live births (14/338). Less than half of the mothers presented their sick newborn at a health facility, however, 82% of these newborns recovered from their illness after treatment.

The most common symptoms or conditions of neonatal illness symptoms perceived by health facility delivered mothers’ in the study area were possibly serious bacterial infections (PSBI) with 95.8% (90/94), localized bacterial infections (LBI) with 43.6% (41/94), low birth weight with 23.4% (22/94), diarrhea with 18.1% (17/94), prematurity with 14.9% (14/94) and jaundice 7.5% (7/94). Among the PSBI perceived symptoms; coughing was recorded with 37.8% (34/90), unable to breastfeed was 34.4% (31/900, fever was 27.8% (25/90), fast-breathing was 20% (18/90), and hypothermia was 14.4% (13/90). Similarly among the LBI symptoms; redness of umbilicus and redness of the eye was recurrent with 26.8% (11/41) and 12.2% (5/41), respectively.

Most of the neonatal illnesses 40.4% (38/94) were perceived with two or more symptoms and a relatively higher proportion of the illness symptoms were perceived at 28^th^ day from birth with 30% (28/94), and at birth with 25% (23/94) **(**S5 and S6 Annexes**)**.

Based on the quality of care during delivery according to essential newborn care components, most newborns with described illness symptoms, 43.6% (41/94) had poor breast-feeding quality, 38.6% (32/83) had poor cord care quality, and 37.7% (43/114) had poor overall newborn care qualities.

### Neonatal mortality

**Fig 2:**
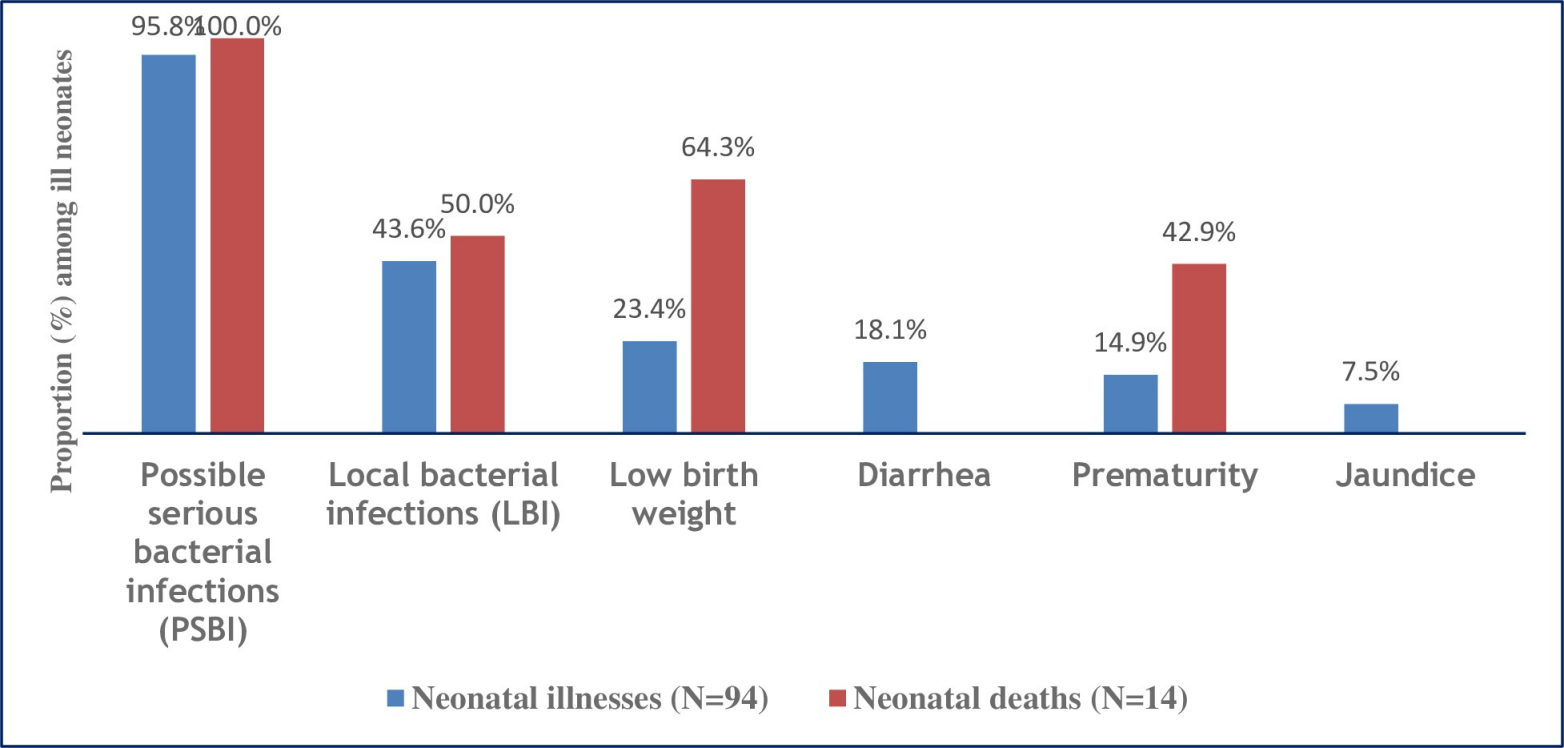
Distribution of neonatal illnesses and proportion of deaths based on symptoms or conditions among the ill neonates in Northwest Ethiopia, March 2019. (*Due to multiple neonatal illnesses*, *the percentage adds up more than 100%*).

**Fig 3:**
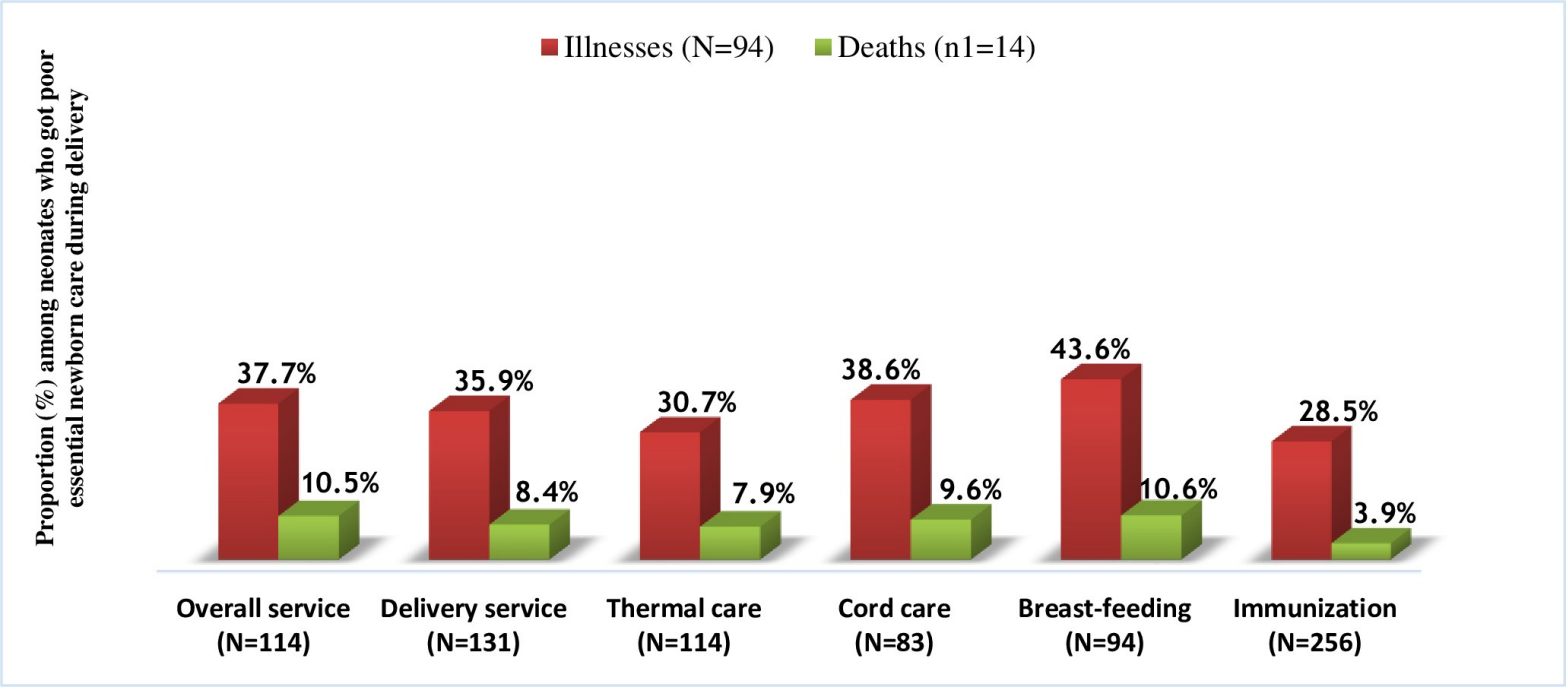
Distribution of neonatal illnesses and deaths among neonates provided with poor quality of essential newborn care during health facility delivery in Northwest Ethiopia, March 2019.

Neonatal mortality rate was 41/1000 live births (14/338). Among the death outcomes, neonates who had PSBI, low birth weight, LBI, and prematurity took the highest proportions with 100% (14/14), 64.3% (9/14), 50% (7/14), and 42.9% (6/14), respectively (Fig 2). Most sick newborns who died were reported to have poor quality of breast-feeding (10.6%, (10/94)), poor overall newborn care quality (10.5%, (12/114)), and poor cord care quality (9.6%, (8/83)) (Fig 3).

### Multilevel models

The ICC calculated based on the null or empty model was significant at 0.29 implying that 29% of the total variation in neonatal illness was attributed due to repeated illnesses. In other words, the correlation between neonates who got illness at birth and the likelihood of having another illness at either 7^th^, 14^th^, and/or 28^th^ day from birth was 0.29. The results of Table 4 showed the null or empty model, model two, when individual level (maternal and neonatal) variables were computed, model three, when facility-level neonatal illness variables were computed, and final model, when individual and health facility level variables were computed together, including both fixed and random effects. The fixed effects of model four showed the associations of both individual and facility characteristics with neonatal illnesses. Hence, model four was the better model to be reported. The multilevel analysis result showed the presence of significant heterogeneity among health facilities. The negative log likelihood ratio and AIC were relatively the smallest in model four.

**Table 4:** Multilevel logistic regression analysis of neonatal illness among health facility deliveries in North Gondar zone, Northwest Ethiopia, 2019.

Variables	Model I ^a^	Model II ^b^	Model III ^c^	Model IV ^d^
**Individual (Neonatal and Maternal) level factors**				
**Number of live children**				
≤ 3 children	-	2.03 (1.1, 3.7)*	-	1.96 (1.1 3.6)*
≥ 4 children		1		1
**ANC attendance at current pregnancy**				
No	-	1.90 (1.1, 3.5)*	-	1.83 (1.1, 3.3)*
Yes		1		1
**Type of pregnancy**				
Single	-	1	-	1
Twins or triplets		2.75 (1.1, 6.9)*		2.43 (1.1, 6.1)*
**Birth length**				
Low	-	8.86 (4.0, 19.5)**	-	7.93 (3.6, 17.3)***
High		1		1
**Breast-feeding quality**				
Poor	-	2.70 (1.6, 4.5)**	-	2.37 (1.4, 4.0)**
Good		1		1
**Health facility level factors**				
**Delivery process quality**				
Poor	-	-	2.34 (1.4, 4.0)*	1.38 (0.8, 2.3)
Good			1	1
**Cord care quality**				
Poor	-	-	1.87 (1.1, 3.2)*	1.42 (0.9, 2.4)
Good			1	1
***Random effects and model comparison***				
ICC	0.29	0.21	0.27	0.20
Log likelihood	-421.69487	-389.99733	-413.1064	-388.29483
AIC	847.3897	796.9947	834.2128	794.5897

a An empty or null model estimating the contextual variation in neonatal morbidity.

b Model simultaneously adjusting for individual-level factors.

c Model simultaneously adjusting for facility-level factors.

d Model simultaneously adjusting for all variables in Model II and Model III.

***p < 0.001, **p < 0.01, and *p < 0.05.

### Multi-collinearity and model fitness

Multi-collinearity effect has been checked by using mean of variation inflation factor (VIF) value. Less than 5 mean VIF value indicates absence of extreme collinearity problem among explanatory variables in the regression model [21]. The negative log likelihood ratio and AIC were relatively lowest in model four. This implies that model four explained the determinants better than either model two or three (Table 4). The statistically non-significant value of the Hosmer-Lemeshow statistics has been (p>0.05) confirmed for the model fitting the data reasonably well. The area under the ROC curve was also 70%.

### Description of determinants

Neonates who come from mothers having a maximum of 3 children were two times more likely to have neonatal illness compared with those who had four and above children (AOR = 1.96; 95% CI = 1.1–3.6). Similarly, having no ANC attendance (AOR = 1.83; 95% CI = 1.1–3.3); having a multiple pregnancy (AOR = 2.43; 95% CI = 1.1–6.1); low birth length (AOR = 7.93; 95% CI = 3.6–17.3), and poor breastfeeding quality (AOR = 2.37; 95% CI = 1.4–4.0) were the determinant factors for neonatal illness after controlling for other factors (Table 4).

## Discussion

This study aimed to determine the prevalence and factors associated with symptoms of newborn illnesses reported by mothers based on the adapted IMCI SIRS definition in Northwest Ethiopia. Consequently, the prevalence of perceived neonatal illness symptoms was 27.8% of all babies born alive. PSBI, LBI, low birth weight, diarrhea, prematurity, and jaundice were the most commonly perceived symptoms or conditions with 95.8%, 43.6%, 23.4%, 18.1%, 14.9%, and 7.5% respectively. Neonatal mortality rate was 41/1000 live births (14/338). Among those newborns who died, 100% (14/14) had PSBI, 64.3% (9/14) had low birth weight, 50% (7/14) had LBI, and 42.9% (6/14) had prematurity. Having a maximum of 3 children, having no antenatal counseling, having a multiple pregnancy, low birth length, and poor breastfeeding quality were the factors associated with perceived neonatal illness symptoms.

This report indicated that neonatal mortality was 41/1000 live births, which is higher than the estimates of sub-Saharan countries [4,5]. The observed high prevalence of neonatal illnesses (27.8%) and neonatal mortality rate compared with the recent finding of the Ethiopian demographic health survey (30/1000 live births) [10] showed that Ethiopia would face difficulty to achieve the goal set by SDGs for all countries to reduce neonatal mortality as low as 12 deaths per 1000 live births or fewer in 2030 [22]. Moreover, limited visiting of newborns by HEW after delivery and low postnatal care attendance at a health facility could also contribute to the situation of high symptom prevalence and high neonatal mortality in the study area.

PSBI’s were the most common sign of neonatal illnesses amongst the study participants (95.8%). This finding was higher than studies done in Gondar University Hospital (77.8% had sepsis) [17] and Burundi (48.6%) [23]. The difference could be due to variations in the number of health facilities included in the study, measurement tool for neonatal morbidities, outcome ascertainment approach (reporting of either prematurity or of birth length) and due to variations in the study population to access health facilities. The previous studies were done in a single health facility, and integrated management of childhood illness guideline was used in this study to measure the symptoms of various newborn illnesses reported by mothers for a better clinical and public health importance [13].

Low birth weight accounted for 23.4% of the neonatal morbidities, which is lower than studies carried out in Ethiopia and Bangladesh [17,24–26], but higher than another study done in Ethiopia [27]. On the other hand, prematurity was recorded to be 14.9% that could be a trait for neonatal illnesses in this study. This is consistent with previous studies in Ethiopia [27], and Burundi [23], but significantly lower than other studies in Ethiopia and other low-income countries [17,25,28–32]. The discrepancy could be attributed to the variations in the utilization of antenatal and newborn care services in health facilities. Health facilities having appropriate antenatal care services can better anticipate and give appropriate counseling to mothers during delivery, and link high risk pregnant newborn babies to health institutions to care for low birth weights and/or prematurity.

Diarrhea was reported in 18.1% of the ill neonates in the study area. This finding was higher than the previous studies in Ethiopia [33,34], but lower than other similar studies in Ethiopia [35,36]. Usually diarrhea is common towards the end of summer and early rainy season, while data of this study was collected during the dry season.

Providing appropriate care during pregnancy is crucial time for the health of the mother and the child. Antenatal care, provides an opportunity to prevent maternal, newborn and child illness, in this study mothers having no antenatal care were nearly 2 times more likely to face neonatal illness compared to their counterparts. This finding is in line with other study done in four poorest Mesoamerican countries (Guatemala, Honduras, Nicaragua, and Mexico) [37]. This could demonstrate that having ANC follow-up creates a good opportunity to get the necessary information about the newborn illness. This is similar to the findings in Jimma [9,38] which reported that ANC improves knowledge on neonatal illness. This could demonstrate that having ANC follow-up could improve the risk identification component of the antenatal care, which creates a good opportunity to identify at risk pregnancies and channel them to appropriate management. Consequently, this could help to ensure the best health outcomes for both the mother and the newborn.

In the current study, mothers who had ≤ 3 living children were more likely to face neonatal illness as compared to those who had ≥ 4 living children. This finding was not supported by a previous study done in Tanzania [39], which explained as children born from larger family size had a higher risk of neonatal illness and delayed for healthcare than their counterparts. The reasons could be due to the improvement of the mothers’ knowledge, skill, experience to manage ill neonates, and reduction of neonatal illness symptoms a head of time. However, it is common that women with more children face higher rates of complications due to the fact that they are often poorer, less likely to have ANC and family planning measures such as child spacing.

Twin or triplet neonates were 2.4 times more likely to develop a neonatal illness as compared to singletons. This might be due to exposure for various delivery complications and to the fact that multiple pregnancies often end preterm, which is associated to higher morbidity. This is also reflected in the fact that length at birth was associated to illness. This could be explained by the fact that newborns with a normal length are more likely to be term babies and without gross intrauterine retardation [40]. Similarly, low birth length newborns are more likely to have immature immune system (low neutrophil storages) and organs that fight infections, and low body temperatures, which increase the risk of illness and death compared to their counterparts [41]. Furthermore, multiple newborns are more likely to be born prematurely with all the consequences of prematurity: reduced length, immune-incompetence, jaundice, feeding difficulties etc.

Besides, this study showed that poor breastfeeding quality was significantly associated with the neonatal illness. Prevalence of illness was high among neonates where poor quality breastfeeding was reported compared to others. This finding is in agreement with previous study findings in Ethiopia [34], and England [39]. Appropriate breastfeeding, including the provision of colostrum, has various benefits in reducing neonatal illnesses due to hypothermia, hypoglycemia, acute respiratory infections, diarrhea, septicemia, and mortality, particularly in the late neonatal period [42].

Knowing the prevalence of neonatal morbidity has various implications for district health office managers, healthcare providers, researchers, and policy makers at large. Different stakeholders would strategically plan for early detection of neonatal illnesses and provision of appropriate healthcare to improve neonatal outcomes in the study area and others similar settings in Ethiopia.

The study has notable strengths. Primary data was used through follow-up (house-to-house survey) starting from their facility delivery until the end of the neonatal period. Neonatal illness symptoms commonly perceived by mothers’ were assessed at birth, 24 hours, 7^th^, 14^th^, and 28^th^ day from birth to record any neonatal illnesses after health facility delivery.

### Limitation

First, the follow-up data was collected within five months (November–March) time, which did not show the annual picture of neonatal illness during summer and winter seasons. Second, as a cross-sectional study, we can infer associations but not causations from our results. Unadjusted neonatal, maternal and facility related factors were considered for this study, which may influence the observed associations. Third, due to lack of clinical equipment’s and supplies, perceived symptoms of neonatal illness data were collected through interview unlike clinical examinations based on the mothers’ perception through house-to-house interviews by using trained midwives and nurses. Fourth, randomly selected newborns including premature births were included in this study. This could affect the association between prematurity and several illness conditions among newborns with described illness symptoms. Fifth, there might be a re-call bias as the mothers’ had to remember the illness symptoms and the care given to their neonates. Lastly, the source and study population were newborns delivered in health facilities, where the majority of women in Ethiopia does not deliver in health institutions.

## Conclusions

This study indicated a high prevalence of neonatal illness symptoms perceived by mothers. Lack of antenatal care, low birth weight, having three or less living children, and poor breastfeeding quality were found to be positively associated with neonatal illnesses. Therefore, early detection, referral and better management of symptoms or conditions with a high mortality, like sepsis and low birth weight are compulsory to save the lives of many neonates. Strengthening the effectiveness of health extension workers to improve antenatal care service utilization and breastfeeding quality of neonates among postpartum women is crucial in Northwest Ethiopia.

## Supporting information

S1 Annex Major categories of neonatal illnesses based on systematic inflammatory response syndrome (SIRS’s) definition. (DOCX)

S2 Annex Data collection tool (English version) to assess the outcome of essential newborn care service utilization among health facility deliveries in Northwest Ethiopia. (DOCX)

S3 Annex Data collection tool (Amharic version) to assess the outcome of essential newborn care service utilization among health facility deliveries in Northwest Ethiopia. (DOCX)

S4 Annex Obstetric characteristics of mothers and delivery assistants in selected health facilities, northwest Ethiopia (N = 338), March 2019. (DOCX)

S5 Annex Distribution of neonatal illnesses with birth dates among health facility deliveries in northwest Ethiopia, March 2019. (DOCX)

S6 Annex Frequency of illness among neonates in health facility deliveries in northwest Ethiopia, March 2019. (DOCX)
