# Supplementary material for: Prevalence of common symptoms of neonatal illness in Northwest Ethiopia: A repeated measure cross-sectional study
Source: PLoS One. 2021 Mar 30;16(3):e0248678. doi: 10.1371/journal.pone.0248678 (PMC8009397; doi:10.1371/journal.pone.0248678)
Supplement: S1 Annex — (DOCX) [file pone.0248678.s001.docx]

**Annex 1:** Major categories of neonatal illnesses based on systematic inflammatory response syndrome (SIRS’s) definition.

| **Categories** | **Definitions** | **Symptoms or Conditions included** |
| --- | --- | --- |
| Possible Serious Bacterial Infection (PSBI) | A clinical syndrome used in the Integrated Management of Childhood Illness (IMCI) package referring to a sick young infant who requires urgent referral to hospital. | - Coughing - Unable to breast-feed - Fever - Fast breathing - Hypothermia - Vomiting - Convulsion |
| Localized Bacterial Infection (LBI) | A clinical syndrome used in the IMCI characterized by an infected umbilicus or a skin infection. | - Redness of umbilicus - Redness of eye - Localized pus - Ear pain |
| Low birth weight (LBW) | Any newborn with a birth weight of less than 2,500 grams | - Low birth weight |
| Prematurity | Any newborn delivered before 37 weeks of gestation | - Preterm births |
| Diarrhea | When a newborn baby passes very runny, liquidly stools, sometimes at an increased frequency or more volume than normal. | - Watery diarrhea - Bloody diarrhea |
| Jaundice | Yellow discoloration of the newborn’s skin, mucous membranes, and the whites of the eyes. | - Yellow discoloration |
