# Supplementary material for: Prevalence of common symptoms of neonatal illness in Northwest Ethiopia: A repeated measure cross-sectional study
Source: PLoS One. 2021 Mar 30;16(3):e0248678. doi: 10.1371/journal.pone.0248678 (PMC8009397; doi:10.1371/journal.pone.0248678)
Supplement: S2 Annex — (DOCX) [file pone.0248678.s002.docx]

**
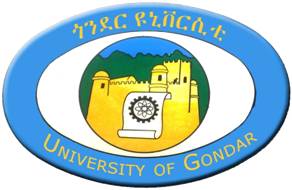
**

**University of Gondar**

**College of Medicine and Health Sciences**

**Institute of Public Health**

**Annex 2፡** Data collection tool (English version) to assess the outcome of essential newborn care service utilization among health facility deliveries in Northwest Ethiopia.

**Health Facility and Respondent’s /Mothers/ Identification:**

**Date of Delivery: -----/-----/2011 E. C.**

|  | **District** | **Kebele** | **Name of Health Facility** | **Respondents Identification** | **Name of data collector** |
| --- | --- | --- | --- | --- | --- |
| **Name** |  |  |  |  |  |
| **ID/Code/** |  |  |  |  |  |

1. **Mothers socio-demographic and socio-economic related questions**

| **S/N** | **Question** | | | **Response/choice/** | | | | | | | | | | | | | | | | | | | | | | |
| --- | --- | --- | --- | --- | --- | --- | --- | --- | --- | --- | --- | --- | --- | --- | --- | --- | --- | --- | --- | --- | --- | --- | --- | --- | --- | --- |
| 1 | Age of mother? | --------------------- years | | | | | | | | | | | | | | | | | | | | | | | | |
| 2 | Marital status? | 1. Married 2. Single | | | | | 1. Divorced 2. Widowed | | | | | | | | | | | | | 1. Separated 2. Living together | | | | | | |
| 3 | Mother’s Education level? | 1. Unable to read and write 2. Able to read and write 3. Grade 1-3 complete 4. Grade 4-6 complete | | | | | | | | | | 1. Grade 7 and 8 complete 2. Grade 9 and 10 complete 3. Grade 11 and 12 complete 4. 12+ | | | | | | | | | | | | | | |
| 4 | Father’s Education level? | 1. Unable to read and write 2. Able to read and write 3. Grade 1-3 complete 4. Grade 4-6 complete | | | | | | | | | | 1. Grade 7 and 8 complete 2. Grade 9 and 10 complete 3. Grade 11 and 12 complete 4. 12+ | | | | | | | | | | | | | | |
| 5 | Mother’s Occupation? | 1. Housewife 2. Merchant 3. Employed (Private) 4. Employed (Government) | | | | | | | | | | | 1. Daily laborer 2. Student 3. House servant 4. Other:-------------------------- | | | | | | | | | | | | | |
| 6 | Father’s Occupation? | 1. Farmer 2. Merchant 3. Employed (Private) 4. Employed (Government) | | | | | | | | | | | 1. Daily laborer 2. Student 3. House servant 4. Other:------------------------ | | | | | | | | | | | | | |
| 7 | Religion? | 1. Orthodox 2. Muslim | | | 1. Catholic 2. Protestant | | | | | | | | | | | | 1. Other:--------------- | | | | | | | | | |
| 8 | Family size? | ------------------ /in number/ | | | | | | | | | | | | | | | | | | | | | | | | |
| 9 | Residence? | 1. Urban | | | | 1. Peri-urban | | | | | | | | | | | | 1. Rural | | | | | | | | |
| 10 | Who is the owner of your living home? | 1. Her/him self | | | | 1. Rent | | | | | | | | 1. Family | | | | | | | | | | 1. Other------ | | |
| 11 | Is the family use Electric/solar energy? | | | | | | | | 1. No | | | | | | | 1. Yes | | | | | | | | | | |
| 12 | Which energy source do you use to prepare food?*(Multiple answers possible)* | | | | | | | | 1. Electric 2. Biogas | | | | | | | 1. Kerosene 2. Wood/coal/dung | | | | | | | | | | |
| 13 | Is the family food mostly prepared within a home? | | | | | | | | 1. No | | | | | | | | | | 1. Yes | | | | | | | |
| 14 | Where is the main source of drinking water for the family? | | | | | | | | | 1. River 2. Dug well | | | | | | | | | 1. Spring water 2. Pipe water | | | | | | | |
| 15 | Which household equipment’s are available at home?*(Multiple answers possible)* | | 1. Radio 2. Mobile | | | | | | | | | | | 1. Refrigerator 2. Television | | | | | | | | | | | | 1. None of them |
| 16 | What type of toilet the family use? | 1. Pour flush latrine 2. Ventilated pit latrine | | | | | | | | | 1. Traditional pit latrine 2. Open well | | | | | | | | | | | | | | 1. Open air | |
| 17 | Where is the toilet facility located? | 1. Own dwelling | | | | | | | | | 1. Own yard | | | | | | | | | | | | | | 1. Elsewhere | |
| 18 | What is the household’s main source of income? | 1. Agriculture 2. Monthly salary | | | | | | 1. Trade 2. Family support | | | | | | | | | | | | | | | 1. Daily labor 2. Other---------- | | | |
| 19 | Does the household have agricultural land? | | | | | | | 1. Yes | | | | | | | | | | | | | | | 1. No **21** | | | |
| 20 | If yes, how much is it? | | | | | | | ---------------------Hectare/s | | | | | | | | | | | | | | | | | | |
| 21 | Does the household have regular salary? | | | | | | | 1. Yes | | | | | | | | | | | | | 1. No **23** | | | | | |
| 22 | If household have regular salary, how much is it? | | | | | | | --------------------- Birr | | | | | | | | | | | | | | | | | | |
| 23 | Does the family have a Bank Account or Microfinance saving account? | | | | | | | 1. No | | | | | | | | | | | | | 1. Yes | | | | | |
| 24 | Does the family have a Health Insurance? | | | | | | | 1. No | | | | | | | | | | | | | 1. Yes | | | | | |
| 25 | Do you have the economic capability to cover all ENC costs of your child? | | | | | | | 1. Yes | | | | | | | | | | | | | 1. No | | | | | |
| 26 | Do you believe that the costs of newborn health care is very high? | | | | | | | 1. Yes | | | | | | | | | | | | | 1. No | | | | | |
| 27 | Does the household have animals? | | | | | | | 1. Yes | | | | | | | | | | | | | | 1. No **29** | | | | |
| 28 | If yes, which animals does the household have? | **Type of animals** | | | | | | | | | | | | | **Number of animals** | | | | | | | | | | | |
|  |  | Domestic animals (cows and oxen) | | | | | | | | | | | | |  | | | | | | | | | | | |
|  |  | Equines (horse, donkey and mules) | | | | | | | | | | | | |  | | | | | | | | | | | |
|  |  | Sheep and goats | | | | | | | | | | | | |  | | | | | | | | | | | |
|  |  | Poultry | | | | | | | | | | | | |  | | | | | | | | | | | |

1. **Mothers health related questions**

| **S/N** | **Question** | **Response/choice/** |
| --- | --- | --- |
| 29 | Age at first marriage? | --------------------- years |
| 30 | Age at first pregnancy? | --------------------- years |
| 31 | Gestation week at current delivery? | --------------------- weeks |
| 32 | How many times did you get pregnant in your life time, including the current pregnancy? | --------------------- /Number/ |
| 33 | How many children did you deliver, including live and still births? | --------------------- Children |
| 34 | Among all your all deliveries, how many of them are still alive? | --------------------- /Number/ |
| 35 | Among all your all deliveries, how many of them were stillbirths? | --------------------- /Number/ |
| 36 | In your lifetime, how many times did you face abortion without your interest/plan? | --------------------- /Number/ |
| 37 | In your lifetime, how many times did you face abortion with your interest/plan? | --------------------- /Number/ |
| 38 | In your lifetime, how many times did you face neonatal deaths? | --------------------- /Number/ |

1. **Mother’s ANC service related questions**

| **S/N** | **Question** | | | | | | | | | | | **Response /choice/** | | | | | | | | | |
| --- | --- | --- | --- | --- | --- | --- | --- | --- | --- | --- | --- | --- | --- | --- | --- | --- | --- | --- | --- | --- | --- |
| 39 | Did you have ANC card? | | | | | | | | | | | 1. No | | | | | | | 1. Yes | | |
| 40 | Have you ever got health education from HEW? | | | | | | | | | | | 1. No | | | | | | | 1. Yes | | |
| 41 | Did you have a family health guiding book in your home? | | | | | | | | | | | 1. No **43** | | | | | | | 1. Yes | | |
| 42 | If you have a family health book, have you ever read or let other to read for you? | | | | | | | | | | | 1. No | | | | | | | 1. Yes | | |
| 43 | Is your pregnancy wanted | | | | 1. Not wanted | | | | 1. Wanted | | | | 1. Not in appropriate time | | | | | | | | |
| 44 | Were you using birth control before the current pregnancy? | | | | | | | | | | | | | | 1. Yes | | | | | | 1. No |
| 45 | Did you attend ANC during your current pregnancy? | | | | | | | | | | | | | | 1. No **53** | | | | | | 1. Yes |
| 46 | If yes, which health care provider did give you the ANC services? | | | | | | 1. HEW 2. Midwife 3. Nurse | | | | | | | | 1. Health Officer 2. Medical Doctor 3. Other /specify/------- | | | | | | |
| 47 | Who encouraged you for the first time to start ANC visit? | | | | | | 1. Myself 2. Husband 3. DA Leader | | | | | | | | 1. 1to5 Leader 2. HEW 3. Other /specify/------- | | | | | | |
| 48 | At which month of your pregnancy did you start your first ANC visit? | | | | | | -------------------------Months | | | | | | | | | | | | | | |
| 49 | Totally, how many ANC visits did you have on the current pregnancy? | | | | | | --------------------------/Number/ | | | | | | | | | | | | | | |
| 50 | Where did you get your ANC services? | | | | | | 1. Health Center 2. House by HEW 3. Private Clinic | | | | | | | | 1. Health Post 2. Hospital 3. Other--------------- | | | | | | |
| 51 | Did you get counseling during your ANC visit? | | | | | | 1. Yes | | | | | | | | 1. No **53** | | | | | | |
| 52 | If yes, which ANC services did you get? | | Advised to deliver at health facility? | | | | | | | | | | | | | 1. Yes | | | | 1. No | |
|  |  |  | Advised to prepare for delivery, informed about the possible challenges and precautions? | | | | | | | | | | | | | 1. Yes | | | | 1. No | |
|  |  |  | Advised on Maternal danger signs during pregnancy? | | | | | | | | | | | | | 1. Yes | | | | 1. No | |
|  |  |  | Advised on Maternal danger signs during and immediately after delivery? | | | | | | | | | | | | | 1. Yes | | | | 1. No | |
|  |  |  | Advised on Neonatal danger signs during and immediately after delivery? | | | | | | | | | | | | | 1. Yes | | | | 1. No | |
|  |  |  | Advised to use Postnatal care /PNC/ services? | | | | | | | | | | | | | 0. Yes | | | | 1. No | |
|  |  |  | Advised on postnatal family planning services? | | | | | | | | | | | | | 1. Yes | | | | 1. No | |
|  |  |  | Advised on feeding practices during pregnancy? | | | | | | | | | | | | | 1. Yes | | | | 1. No | |
|  |  |  | Breastfeeding /Neonatal feeding practices/? | | | | | | | | | | | | | 1. Yes | | | | 1. No | |
|  |  |  | Advised to visit health facilities while facing danger signs during pregnancy and delivery? | | | | | | | | | | | | | 1. Yes | | | | 1. No | |
|  |  |  | Counseling on HIV testing? | | | | | | | | | | | | | 1. Yes | | | | 1. No | |
|  |  |  | Advised to test and take Anthelmintics? | | | | | | | | | | | | | 1. Yes | | | | 1. No | |
|  |  |  | Advised to take Iron Folate to prevent Anemia? | | | | | | | | | | | | | 1. Yes | | | | 2. No | |
|  |  |  | Advised to use ITN to prevent Mosquito bites? | | | | | | | | | | | | | 1. Yes | | | | 2. No | |
|  |  |  | Advised to take Tetanus Antitoxin /TT/? | | | | | | | | | | | | | 1. Yes | | | | 2. No | |
| 53 | How many times did you take Tetanus Antitoxin /TT/? | | | | | | | | | | 1. Not taken 2. TT-1 | | | | | | | | 1. TT-2 | | |
| 54 | Advised to take Iron Folate to prevent Anemia? | | | | | | | | | | 1. No | | | | | | | | 1. Yes | | |
| 55 | Advised to test HIV during this pregnancy? | | | | | | | | | | 1. No | | | | | | | | 1. Yes | | |
| 56 | Advised to take anthelmintic to prevent gut parasites during this pregnancy? | | | | | | | | | | 1. No | | | | | | | | 1. Yes | | |
| 57 | Did you use bed nets during this pregnancy? | | | | | | | | | | 1. Yes | | | | | | | | 1. No | | |
| 58 | Did you face illness during this pregnancy? | | | | | | | | | | 1. Yes | | | | | | | | 1. No  **60** | | |
| 59 | If yes, what type of illness did you face? | Severe headache? | | | | | | | | | 1. No | | | | | | | | 1. Yes | | |
|  |  | Uterine bleeding? | | | | | | | | | 1. No | | | | | | | | 1. Yes | | |
|  |  | Shivering? | | | | | | | | | 1. No | | | | | | | | 1. Yes | | |
|  |  | Edema at hand, face or whole body? | | | | | | | | | 1. No | | | | | | | | 1. Yes | | |
|  |  | Severe anemia /circling/? | | | | | | | | | 1. No | | | | | | | | 1. Yes | | |
|  |  | Febrile /increased body temperature/? | | | | | | | | | 1. No | | | | | | | | 1. Yes | | |
|  |  | Hypothermia /decreased body temperature/? | | | | | | | | | 1. No | | | | | | | | 1. Yes | | |
|  |  | Heavy uterine bleeding? | | | | | | | | | 1. No | | | | | | | | 1. Yes | | |
|  |  | Increased blood pressure /BP/? | | | | | | | | | 1. No | | | | | | | | 1. Yes | | |
|  |  | Convulsion? | | | | | | | | | 1. No | | | | | | | | 1. Yes | | |
|  |  | Unable to control urination or defecation? | | | | | | | | | 1. No | | | | | | | | 1. Yes | | |
|  |  | Foul smell uterine fluid? | | | | | | | | | 1. No | | | | | | | | 1. Yes | | |
|  |  | Diabetes | | | | | | | | | 1. No | | | | | | | | 1. Yes | | |
| 60 | Type of pregnancy? | | | | | | | 1. Single | | | | | | 1. Twins | | | | | 1. Three and above | | |
| 61 | Sex of the newborn | | | | | | | 1. Male | | | | | | 1. Female | | | | | | | |
| 62 | Outcome of this pregnancy? | | | | | 1. Term birth 2. Stillbirth 3. Died immediately after birth | | | | | | | | | | | 1. Induced abortion 2. Natural abortion | | | | |
| 63 | Newborn presentation during delivery? | | | | | | | | | 1. Head  2. Sitting  3. Side-lying | | | | | | | | 4. I do not know  5. Other-------------- | | | |
| 64 | Type of delivery? | | | | | | | | | 1. Vaginal/Normal  2. Stich  3. Instrumental | | | | | | | | 4. CS  5. Episiotomy | | | |
| 65 | Who assisted you during delivery? | | | | | | | | | 1. Medical Doctor  2. Nurse | | | | | | | | 3. Midwife Nurse  4. Health Officer | | | |
| 66 | What was the sex of the health professional who assisted you during delivery? | | | | | | | | | 1. Female | | | | | | | | 2. Male | | | |
| 67 | Did the delivery assistant washed her/his hands before assisting you during delivery? | | | | | | | | | 1. No | | | | | | | | 2. Yes | | | |
| 68 | Did the delivery assistant cleaned the perineum area of the newborn after birth? | | | | | | | | | 1. No | | | | | | | | 2. Yes | | | |
| 69 | Did you get counseling services about neonatal danger signs during delivery from the delivery assistant? | | | | | | | | | 0. Yes | | | | | | | | 1. No | | | |
| 70 | If yes, on which neonatal danger signs did you get counseling? | | | Unable to breastfed | | | | | | 0. Yes | | | | | | | | 1. No | | | |
|  |  |  |  | Fever? | | | | | | 0. Yes | | | | | | | | 1. No | | | |
|  |  |  |  | Hypothermia | | | | | | 0. Yes | | | | | | | | 1. No | | | |
|  |  |  |  | Convulsion | | | | | | 0. Yes | | | | | | | | 1. No | | | |
|  |  |  |  | Fast breathing | | | | | | 0. Yes | | | | | | | | 1. No | | | |
|  |  |  |  | Shivering | | | | | | 0. Yes | | | | | | | | 1. No | | | |
|  |  |  |  | Bloating | | | | | | 0. Yes | | | | | | | | 1. No | | | |
|  |  |  |  | Bloody diarrhea | | | | | | 0. Yes | | | | | | | | 1. No | | | |
|  |  |  |  | Watery diarrhea | | | | | | 0. Yes | | | | | | | | 1. No | | | |
|  |  |  |  | Coughing | | | | | | 0. Yes | | | | | | | | 1. No | | | |
|  |  |  |  | Vomiting all contents | | | | | | 0. Yes | | | | | | | | 1. No | | | |
|  |  |  |  | Redness & bleeding at the umbilical cord? | | | | | | 0. Yes | | | | | | | | 1. No | | | |
|  |  |  |  | Yellow discoloration of hands and legs? | | | | | | 0. Yes | | | | | | | | 1. No | | | |
|  |  |  |  | Redness of eye | | | | | | 0. Yes | | | | | | | | 1. No | | | |
|  |  |  |  | Ear pain | | | | | | 0. Yes | | | | | | | | 1. No | | | |

**iv. Essential Newborn Care Outcome**

| **ID** | **Question** | | **Responses /Fill blank spaces and circle Yes/No questions clearly/** | | | | | | | | | | | | | | | | | | | | | | |
| --- | --- | --- | --- | --- | --- | --- | --- | --- | --- | --- | --- | --- | --- | --- | --- | --- | --- | --- | --- | --- | --- | --- | --- | --- | --- |
|  |  |  | **At birth** | | | | | **24 hrs.** | | | | **Day 7** | | | | | **Day 14** | | | | | | **Day 28** | |  |
| 71 | Birth weight? | | ---------gram | | | | | -------gram | | | | ------gram | | | | | --------gram | | | | | | ---------gram | |  |
| 72 | Newborn height? | | ---------cm | | | | | ---------cm | | | | --------cm | | | | | ---------cm | | | | | | ---------cm | |  |
| 73 | Neonatal mortality? **93** | | Yes | | | No | | Yes | | No | | Yes | | No | | | Yes | | No | | | | Yes | No |  |
| 74 | Neonatal illness? **93** | | Yes | | | No | | Yes | | No | | Yes | | No | | | Yes | | No | | | | Yes | No |  |
| 75 | If **yes**, which one do you face? | Unable to breastfed | Yes | | | No | | Yes | | No | | Yes | | No | | | Yes | | | No | | | Yes | No |  |
|  |  | Fever? | Yes | | | No | | Yes | | No | | Yes | | No | | | Yes | | | No | | | Yes | No |  |
|  |  | Hypothermia | Yes | | | No | | Yes | | No | | Yes | | No | | | Yes | | | No | | | Yes | No |  |
|  |  | Convulsion | Yes | | | No | | Yes | | No | | Yes | | No | | | Yes | | | No | | | Yes | No |  |
|  |  | Fast breathing | Yes | | | No | | Yes | | No | | Yes | | No | | | Yes | | | No | | | Yes | No |  |
|  |  | Shivering | Yes | | | No | | Yes | | No | | Yes | | No | | | Yes | | | No | | | Yes | No |  |
|  |  | Bloating | Yes | | | No | | Yes | | No | | Yes | | No | | | Yes | | | No | | | Yes | No |  |
|  |  | Bloody diarrhea | Yes | | | No | | Yes | | No | | Yes | | No | | | Yes | | | No | | | Yes | No |  |
|  |  | Watery diarrhea | Yes | | | No | | Yes | | No | | Yes | | No | | | Yes | | | No | | | Yes | No |  |
|  |  | Coughing | Yes | | | No | | Yes | | No | | Yes | | No | | | Yes | | | No | | | Yes | No |  |
|  |  | Vomiting all contents | Yes | | | No | | Yes | | No | | Yes | | No | | | Yes | | | No | | | Yes | No |  |
|  |  | Redness & bleeding at the umbilical cord? | Yes | | | No | | Yes | | No | | Yes | | No | | | Yes | | | No | | | Yes | No |  |
|  |  | Yellow discoloration of hands and legs? | Yes | | | No | | Yes | | No | | Yes | | No | | | Yes | | | No | | | Yes | No |  |
|  |  | Redness of eye | Yes | | | No | | Yes | | No | | Yes | | No | | | Yes | | | No | | | Yes | No |  |
|  |  | Ear pain | Yes | | | No | | Yes | | No | | Yes | | No | | | Yes | | | No | | | Yes | No |  |
|  |  | Other:------------ | Yes | | | No | | Yes | | No | | Yes | | No | | | Yes | | | No | | | Yes | No |  |
| 76 | After how long did you leave the delivery health facility to home? | | | | | | | | | | | | ----------days-----------hours | | | | | | | | | | | |  |
| 77 | Did the newborn get health care during any neonatal illness? | | | | | | | | | | | | 1. No **98** | | | | | | | | 1. Yes | | | |  |
| 78 | If yes, after how long did he/she get health care? | | | | | | | | | | | | -----------days**-----------hours** | | | | | | | | | | | |  |
| 79 | Where did you get neonatal health care?(Multiple answers possible) | | | 1. Traditional healers 2. Government hospital 3. Health Center 4. Health Post | | | | | | | | | | | | 1. Private Clinic 2. Pharmacy/drug store 3. Homemade drug 4. Other---------------------- | | | | | | | | |  |
| 80 | What was the progress after neonatal health care? | | | | | | | | 1. Recovered | | | | | | 1. Not recovered | | | | | | | | 1. Died | |  |
| 81 | Did the newborn attend postnatal care? | | | | | | | | 1. No | | | | | | | 1. Yes | | | | | | | | |  |
| 82 | Have you been visited by HEW after delivery? | | | | | | | | 1. Yes | | | | | | | 2. No **101** | | | | | | | | |  |
| 83 | If yes, after how long did the HEW visited you? | | | | | | | | **----------days**-----------hours | | | | | | | | | | | | | | | |  |
| 84 | Due to the above neonatal health problems, at least how many times did you visited health facilities until 28^th^ day? | | | | | | | | ---------------------/Number/ | | | | | | | | | | | | | | | |  |
| 85 | Neonatal status at 28^th^ day from birth? | | | | 1. Alive safely | | | | | | 1. Alive with disabilities | | | | | | | | | | | 1. Died | | |  |
| 86 | If disabled, when did you think this disability occurred? | | | | | | 1. Congenital 2. During delivery | | | | | | | | | 1. After delivery 2. I do not know | | | | | | | | |  |
| 87 | Did you have a health facility nearby to be visited whenever the newborn get sick? | | | | | | 1. Yes | | | | | | | | | 2. No | | | | | | | | |  |
| 88 | Is the road from your home to health facility accessible for vehicles? | | | | | | 0. Yes | | | | | | | | | 1. No | | | | | | | | |  |
| 89 | How far is your home from the nearest health center or how long does it take? | | | | | | ----------------Kilo meters  **----------------Minutes**-------------hours | | | | | | | | | | | | | | | | | |  |
| 90 | What type of transport system did you use to go to the nearest health center? (Multiple answers possible) | | | | | | 1. On foot  2. Cultural carrying methods | | | | | | | | | | | 3. Horse/Mule  4. Cart  5. Vehicles | | | | | | |  |
| 91 | What type of road did you use to go to the nearest health center? | | | | | | 1. No road  2. Road accessible in al weather conditions | | | | | | | | | | | 3. Road accessible during dry season  4. Asphalt road | | | | | | |  |
| 92 | How long would you take on average to reach to the nearest health center by using the available transport system? | | | | | | **----------------Minutes**-------------hours | | | | | | | | | | | | | | | | | |  |
| 93 | How far is your home from the nearest health post or how long does it take? | | | | | | ----------------Kilo meters | | | | | | | | | | | | | | | | | |  |
| 94 | How long would you take on average to reach to the nearest health post by using the available transport system? | | | | | | **----------------Minutes**-------------hours | | | | | | | | | | | | | | | | | |  |
| 95 | Generally, did you think that getting neonatal health care is difficult? | | | | | | 1. Yes | | | | | | | | | | | 2. No | | | | | | |  |
