# Supplementary material for: Prevalence of common symptoms of neonatal illness in Northwest Ethiopia: A repeated measure cross-sectional study
Source: PLoS One. 2021 Mar 30;16(3):e0248678. doi: 10.1371/journal.pone.0248678 (PMC8009397; doi:10.1371/journal.pone.0248678)
Supplement: S3 Annex — (DOCX) [file pone.0248678.s003.docx]

***We thank you for providing the information!!!***

**
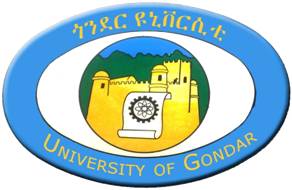
**

**በጎንደር ዩኒቨርሲቲ**

**ህክምናና ጤና ሳይንስ ኮሌጅ**

**ህብረተሰብ ጤና አጠባበቅ ተቋም**

**Annex 3፡** Data collection tool (Amharic version) to assess the outcome of essential newborn care service utilization among health facility deliveries in Northwest Ethiopia.

**የጨቅላ ህፃናት ህክምና አገልግሎት ውጤትን ለመለካት የቀረበ የምርምር ንድፈ-ሃሳብ መጠይቅ፡፡**

**የጥናቱ ተሳታፊዎች መረጃ እና የስምምነት ማስረጃ ቅፅ**

**የጤና ተቋሙና የመላሸዋ የማንነት መለያ፡፡ የወለዱበት ቀን፡ -------/-------/2011 ዓ/ም**

|  | **ወረዳ** | **ቀበሌ** | **የጤና ተቋም ስም** | **የተጠያቂ መለያ** | **የመረጃ ሰብሳቢው ስም** |
| --- | --- | --- | --- | --- | --- |
| **ስም** |  |  |  |  |  |
| **አይዲ/ኮድ** |  |  |  |  |  |

1. **የእናቶች ማህበራዊና ኢኮኖሚያዊ መጠይቆች፤**

| **ተ/ቁ** | **ጥያቄ** | | | | | | **ምላሽ/ምርጫ** | | | | | | | | | | | | | | | |
| --- | --- | --- | --- | --- | --- | --- | --- | --- | --- | --- | --- | --- | --- | --- | --- | --- | --- | --- | --- | --- | --- | --- |
| 1 | የእናት ዕድሜ? | | | ------------- ዓመት | | | | | | | | | | | | | | | | | | |
| 2 | የጋብቻ ሁኔታ? | | | 1. ባለትዳር 2. ያላገባች | 1. የፈታች 2. ባሏ የሞተባት | | | | | | | | 1. ተለያይተው የሚኖሩ 2. ሳይጋቡ አብረው የሚኖሩ | | | | | | | | | |
| 3 | የእናት የትምህርት ደረጃ | | | 1. ማንበብና መፃፍ የማትችል 2. ማንበብና መፃፍ የምትችል 3. ከ1-3ኛ ክፍል ያጠናቀቀች 4. ከ4-6ኛ ክፍል ያጠናቀቀች | | | | | | | | 1. 7 እና 8ኛ ክፍል ያጠናቀቀች 2. 9 እና 10ኛ ክፍል ያጠናቀቀች 3. 11 እና 12ኛ ክፍል ያጠናቀቀች 4. ከ12ኛ ክፍል በላይ የተማረች | | | | | | | | | | |
| 4 | የአባት የትምህርት ደረጃ | | | 1. ማንበብና መፃፍ የማትችል 2. ማንበብና መፃፍ የምትችል 3. ከ1-3ኛ ክፍል ያጠናቀቀች 4. ከ4-6ኛ ክፍል ያጠናቀቀች | | | | | | | | 1. 7 እና 8ኛ ክፍል ያጠናቀቀች 2. 9 እና 10ኛ ክፍል ያጠናቀቀች 3. 11 እና 12ኛ ክፍል ያጠናቀቀች 4. ከ12ኛ ክፍል በላይ የተማረች | | | | | | | | | | |
| 5 | የእናት ሙያ | | | 1. የቤት እመቤት 2. ነጋዴ 3. ከግል ተቀጥረው የሚሰሩ 4. ከመንግሰት ተቀጥረው የሚሰሩ | | | | | | | | | | 1. የቀን ሰራተኛ 2. ተማሪ 3. የቤት ሰራተኛ 4. ሌላ፡---------------------------- | | | | | | | | |
| 6 | የአባት ሙያ | | | 1. ገበሬ 2. ነጋዴ 3. ከግል ተቀጥረው የሚሰሩ 4. ከመንግሰት ተቀጥረው የሚሰሩ | | | | | | | | | | 1. የቀን ሰራተኛ 2. ተማሪ 3. የቤት ሰራተኛ 4. ሌላ፡---------------------------- | | | | | | | | |
| 7 | ሀይማኖት | | | 1. ኦርቶዶክስ 2. ሙስሊም | | | | 1. ካቶሊክ 2. ፕሮቴስታንት | | | | | | | | | 1. ሌላ:--------------- | | | | | |
| 8 | የቤተሰብ ብዛት | | | --------------------/በቁጥር/ | | | | | | | | | | | | | | | | | | |
| 9 | የመኖሪያ አካባቢ | | | 1. ከተማ | | | | | 1. ከተማ-ቀመስ | | | | | | | | 1. ገጠር | | | | | |
| 10 | አሁን የሚኖሩበት ቤት የማን ነው? | | | 1. የግላቸው 2. ተከራይተው | | | | | 1. የቤተሰብ/ዘመድ | | | | | | | | 1. ሌላ ከሆነ ይጥቀሱ---------- | | | | | |
| 11 | ይህ ቤተሰብ የኤሌክትሪክ/ሶላር ተጠቃሚ ነው? | | | | | | | | 1. አይደለም | | | | | | | | | 1. አዎ | | | | |
| 12 | ምግብ ለማብሰል ምን ዓይነት ኃይል ይጠቀማሉ?  *(ከአንድ በላይ መልስ መመለስ ይቻላል)* | | | | | | | | 1. ኤሌክትሪክ 2. ባዮጋዝ | | | | | | | | 1. ኬሮሲን 2. እንጨት/ከሰል/ኩበት | | | | | |
| 13 | ምግብ አብዛኛውን የሚበስለው ቤት ውስጥ ነው? | | | | | | | | 1. አይደለም | | | | | | | | | 1. አዎ | | | | |
| 14 | ይህ ቤተሰብ የሚጠጣ ውሃ በብዛት ከየት ያገኛል? | | | | | | | | 1. ከወንዝ 2. የጉድጓድ ውሃ | | | | | | | | | 1. የምንጭ ውሃ 2. የቧንቧ ውሃ | | | | |
| 15 | ከቤት ውስጥ ከሚከተሉት ቁሳቁሶች የትኞቹ ይገኛሉ?*(ከአንድ በላይ መልስ መመለስ ይቻላል)* | | | | | | | | 1. ሬዲዮን 2. ሞባይል | | | | | | 1. ፍሪጅ 2. ቴሌቪዥን | | | | | | | 1. ምንም የለም |
| 16 | ቤተሰቡ ምን ዓይነት ሽንት ቤት ይጠቀማል? | | 1. የውሃ/ፍላሽ 2. ማስተንፈሻ ያለው | | | | | | 1. ማስተንፈሻ የሌለው 2. ክፍት ጉድጓድ | | | | | | | | | | 1. ከውጭ/ሜዳ ላይ በመፀዳዳት | | | |
| 17 | ሽንት ቤቱ ያለው የት ነው? | | 1. ከቤታቸው ግቢ | | | | | | 1. በማሳቸው | | | | | | | | | | 1. ከዚህ ውጪ | | | |
| 18 | የቤተሰቡ ዋና የገቢ ምንጭ ምንድን ነው? | 1. ግብርና 2. ወርሃዊ/ደሞዝተኛ | | | | 1. ንግድ 2. በቤተሰብ/ዘመድ ድጋፍ | | | | | | | | | | | | 1. የቀን ሰራተኛ 2. ሌላ ካለ ይጠቀስ---- | | | | |
| 19 | ቤተሰቡ የእርሻ መሬት አለው? | | | | | | | | | 1. አዎ | | | | | | | | | 1. የለውም 2**1** | | | |
| 20 | አዎ ካሉ ምን ያህል ነው? | | | | | | | | | -------------------ሄክታር | | | | | | | | | | | | |
| 21 | ቤተሰበ ቋሚ የሆነ የወር ገቢ አለው? | | | | | | | | | 1. አዎ | | | | | | | | | 1. የለኝም **23** | | | |
| 22 | አዎ ከሆነ አማካይ የወር ገቢው ምን ያህል ነው? | | | | | | | | | ------------------ ብር | | | | | | | | | | | | |
| 23 | ቤተሰቡ የባንክ አካውንት ወይም የማይክሮ ፋይናንስ ቁጠባ አለው? | | | | | | | | | 1. የለውም | | | | | | | | | | | 1. አለው | |
| 24 | ይህ ቤተሰብ የጤና መድህን /ኢንሹራንስ/ ተጠቃሚ ነው? | | | | | | | | | 1. አይደለም | | | | | | | | | | 1. ነው | | |
| 25 | የጨቅላ ልጅዎን ማነኛውንም የህክምና ወጪ የሚሸፍን የኢኮኖሚ አቅም አለዎት? | | | | | | | | | 0. አዎ | | | | | | | | | | 1. የለኝም | | |
| 26 | በእርስዎ ግንዛቤ የጨቅላ ህፃናት የህክምና ወጪ በጣም ከፍተኛ ነው ብለው ያምናሉ? | | | | | | | | | 1. አዎ | | | | | | | | | | 1. አይደለም | | |
| 27 | ቤተሰቡ እንስሳት አሉት? | | | 1. አዎ | | | | | | | 1. የለውም **29** | | | | | | | | | | | |
| 28 | አዎ ከሆነ የትኞቹ እንስሳት አሉት? | | | **የእንስሳት ዓይነት** | | | | | | | | | | | | **ብዛት/ቁጥር/** | | | | | | |
|  |  |  |  | የዳልጋ ከብቶች (ላም እና በሬ) | | | | | | | | | | | |  | | | | | | |
|  |  |  |  | የጋማ ከብቶች (አህያ፤ ፈረስ እና በቅሎ) | | | | | | | | | | | |  | | | | | | |
|  |  |  |  | በግ እና ፍየል | | | | | | | | | | | |  | | | | | | |
|  |  |  |  | ዶሮዎች | | | | | | | | | | | |  | | | | | | |

1. **የእናቶች ጤና-ነክ መጠይቆች፤**

| **ተ/ቁ** | **ጥያቄ** | **ምላሽ/ምርጫ** | |
| --- | --- | --- | --- |
| 29 | ለመጀመሪያ ጊዜ ሲያገቡ ዕድሜዎ ምን ያህል ነበር? | ------------- ዓመት | |
| 30 | ለመጀመሪያ ጊዜ ሲያረግዙ ዕድሜዎ ምን ያህል ነበር? | ------------- ዓመት | |
| 31 | አሁን በስንተኛው የእርግዝና ሳምንታት ወለዱ? | ------------- ሳምንታት | |
| 32 | በህይወት ዘመንዎ የአሁኑን ጨምሮ ምን ያህል ጊዜ አርግዘው ያውቃሉ? | | ------------ /በቁጥር/ |
| 33 | በህይወት ዘመንዎ ምን ያህል ልጆችን ወልደዋል /በህይዎት ያሉትንና ሞተው የተወለዱትን ጨምሮ/? | | ------------ ልጆች |
| 34 | በህይወት ዘመንዎ ከወለዷቸው ልጆች መካከል ምን ያህሉ አሁን በህይወት አሉ? | | ------------ /በቁጥር/ |
| 35 | በህይወት ዘመንዎ ከወለዷቸው ልጆች መካከል ምን ያህሉ ሞተው ተወለዱ? | | ------------ /በቁጥር/ |
| 36 | በህይወት ዘመንዎ ምን ያህል ጊዜ ሳይፈልጉ አስወርዶዎት ያውቃል? | | ------------ /በቁጥር/ |
| 37 | በህይወት ዘመንዎ ምን ያህል ጊዜ ፈልገው አስወርደው ያውቃል? | | ------------ /በቁጥር/ |
| 38 | በህይወት ዘመንዎ ልጅዎን ከወለዱ በኃላ 28 ቀን ሳይሞላቸው የሞቱ/የጠፉ/ ልጆችዎ ምን ያህል ይሆናሉ? | | ------------ /በቁጥር/ |

1. **እናቶች በእርግዝና ወቅት ስለተሰጣቸው ቅድመ-ወሊድ ህክምና አገልግሎቶችና የእርግዝና ውጤቶች፤**

| **ተ/ቁ** | **ጥያቄ** | | | | | | | | | | | | | **ምላሽ/ምርጫ** | | | | | | | | | | | | | | | | | | |
| --- | --- | --- | --- | --- | --- | --- | --- | --- | --- | --- | --- | --- | --- | --- | --- | --- | --- | --- | --- | --- | --- | --- | --- | --- | --- | --- | --- | --- | --- | --- | --- | --- |
| 39 | የቅድመ-ወሊድ ህክምና መከታተያ ካርድ አለዎት? (ካርዱን ያሳዩ) | | | | | | | | | | | | | | | | 1. የለኝም | | | | | | | | | | | 1. አለኝ | | | | |
| 40 | ከጤና ኤክስቴንሽን ባለሙያ የጤና ትምህርት አግኝተው ያውቃሉ? | | | | | | | | | | | | | | | | 1. አላውቅም | | | | | | | | | | | | 1. አውቃለሁ | | | |
| 41 | በቤትዎ ውስጥ የቤተሰብ ጤና መመሪያ መፅሀፍ አሎት? (ካለ ያሳዩ) | | | | | | | | | | | | | | | | 1. የለኝም **43** | | | | | | | | | | | | | | | 1. አለ |
| 42 | የቤተሰብ የጤና መመሪያው ካለ አንብበው/አስነብበውት ያውቃሉ? | | | | | | | | | | | | | | | | 1. አላውቅም | | | | | | | | | | | | 1. አውቃለሁ | | | |
| 43 | ያሁኑ እርግዝናዎ የተፈለገ ነው? | | | | | 1. ያልተፈለገ ነበር | | | | | 1. የተፈለገ ነው | | | | | | | | | | | 1. ጊዜውን ያልጠበቀ ነው | | | | | | | | | | |
| 44 | ከአሁኑ እርግዝና በፊት የወሊድ መቆጣጠሪያ ይጠቀሙ ነበር? | | | | | | | | | | | | | | | 1. አዎ | | | | | | | | | | | | | | | 1. የለም | |
| 45 | በዚህ እርግዝና የቅድመ-ወሊድ ህክምና ክትትል አድርገው ነበር? | | | | | | | | | | | | | | | 1. የለም **53** | | | | | | | | | | | | | | | 1. አዎ | |
| 46 | **አዎ** ከሆነ በዚህ ወቅት ለቅድመ-ወሊድ ክትትል ያደረገልዎት ባለሙያ ምን ነበር? | | | | | | 1. የጤና ኤክስቴንሽን 2. አዋላጅ ነርስ 3. ነርስ | | | | | | | | | | | | | | 1. ጤና መኮንን 2. ዶክተር 3. ሌላ----------------- | | | | | | | | | | | |
| 47 | የቅድመ-ወሊድ ህክምና ክትትል አድርገው ከሆነ መጀመሪያ የገፋፋዎት ማን ነበር? | | | | | | 1. በራሴ ተነሳሽነት 2. ባለቤቴ 3. የልማት ቡድን መሪ | | | | | | | | | | | | | | 1. የ1ለ5 መሪ 2. የጤና ኤክስቴንሽን 3. ሌላ ---------------- | | | | | | | | | | | |
| 48 | የስንት ወር ነፍሰ-ጡር ሆነው ነበር ለዚህ እርግዝና የመጀመሪያውን የቅድመ-ወሊድ ክትትል/ምርመራ/ ያደረጉት? | | | | | | | | | | | | | | | | | | | | | | | | -----------ወራት | | | | | | | |
| 49 | በአጠቃላይ በዚህ እርግዝና ምን ያህል ጊዜ የቅድመ-ወሊድ ህክምና ክትትል አድርገዋል? | | | | | | | | | | | | | | | | | | | | | | | | ------------/በቁጥር/ | | | | | | | |
| 50 | የቅድመ-ወሊድ ህክምና ክትትል ያደረጉት የት ነበር? | | | | 1. ጤና ጣቢያ 2. በቤቴ በጤና ኤክስቴንሽን ባለሙያ 3. በግል ክሊኒክ | | | | | | | | | | | | | | | | | | | 1. በጤና ኬላ 2. በሆስፒታል 3. ሌላ-------- | | | | | | | | |
| 51 | በቅድመ-ወሊድ ክትትል ወቅት የምክር አገልግሎቶች አግኝተዋል? | | | | | | | | | | | | | | 1. አዎ | | | | | | | | 1. የለም **53** | | | | | | | | | |
| 52 | **አዎ** ከሆነ ስለየትኞቹ አገልግሎቶች በቅድመ-ወሊድ ወቅት ምክር አገኙ? | በጤና ተቋም እንዲወልዱ ምክር አግኝተዋል? | | | | | | | | | | | | | | | | 1. አዎ | | | | | | | | | | 1. የለም | | | | |
|  |  | ለወሊድ ስለመዘጋጀትና ሊከሰቱ ስለሚችሉ ችግሮች ሊወሰድ ስለሚገባቸው ጥንቃቄዎች? | | | | | | | | | | | | | | | | 1. አዎ | | | | | | | | | | 1. የለም | | | | |
|  |  | በእርግዝና ወቅት እናት ላይ ስለሚከሰቱ አደገኛ የህመም ምልክቶች? | | | | | | | | | | | | | | | | 1. አዎ | | | | | | | | | | 1. የለም | | | | |
|  |  | በወሊድና ወዲያውኑ ከወሊድ በኃላ እናት ላይ ስለሚከሰቱ አደገኛ የህመም ምልክቶች? | | | | | | | | | | | | | | | | 1. አዎ | | | | | | | | | | 1. የለም | | | | |
|  |  | በወሊድና ወዲያውኑ ከወሊድ በኃላ ጨቅላ ህፃን ላይ ስለሚከሰቱ አደገኛ የህመም ምልክቶች? | | | | | | | | | | | | | | | | 1. አዎ | | | | | | | | | | 1. የለም | | | | |
|  |  | ስለድህረ-ወሊድ ህክምና ክትትል የምክር አገልግሎት? | | | | | | | | | | | | | | | | 1. አዎ | | | | | | | | | | 1. የለም | | | | |
|  |  | ስለድህረ-ወሊድ ቤተሰብ ምጣኔ የምክር አገልግሎት? | | | | | | | | | | | | | | | | 1. አዎ | | | | | | | | | | 1. የለም | | | | |
|  |  | በእርግዝና ወቅት ስለሚደረግ የአመጋገብ ሁኔታ? | | | | | | | | | | | | | | | | 1. አዎ | | | | | | | | | | 1. የለም | | | | |
|  |  | ስለሚወለደው ህፃን አመጋገብ (ጡት ማጥባት)? | | | | | | | | | | | | | | | | 1. አዎ | | | | | | | | | | 1. የለም | | | | |
|  |  | በእርግዝናና በወሊድ ወቅት ለሚከሰቱ አደገኛ የጤና ችግሮች የት እንደሚሄዱ ምክር አግኝተዋል? | | | | | | | | | | | | | | | | 1. አዎ | | | | | | | | | | 1. የለም | | | | |
|  |  | በዚህ እርግዝና HIV እንዲመረመሩ ምክር አግኝተዋል? | | | | | | | | | | | | | | | | 0. አዎ | | | | | | | | | | 1. የለም | | | | |
|  |  | በዚህ እርግዝና ወቅት የአንጀት ትላትልን ለማስወገድ መድሃኒት እንዲወሰዱ ምክር አግኝተዋል? | | | | | | | | | | | | | | | | 0. አዎ | | | | | | | | | | 1. የለም | | | | |
|  |  | በዚሀ እርግዝና የደም ማነስ መከላከያ መድሃኒት (አይረን ፎሌት) እንዲወሰዱ ምክር አግኝተዋል? | | | | | | | | | | | | | | | | 0. አዎ | | | | | | | | | | 1. የለም | | | | |
|  |  | በዚህ እርግዝና ወቅት የአልጋ አጎበር ተጠቅመዋል? | | | | | | | | | | | | | | | | 1. አዎ | | | | | | | | | | 1. የለም | | | | |
|  |  | በዚህ እርግዝና በክንድ የሚወጋ የመንጋጋ ቆልፍ መከላከያ ክትባት እንዲከተቡ ምክር አግኝተዋል? | | | | | | | | | | | | | | | | 0. አዎ | | | | | | | | | | 1. የለም | | | | |
| 53 | በዚህ እርግዝና በክንድ የሚወጋ የመንጋጋ ቆልፍ መከላከያ ክትባት ስንት ጊዜ ተከትበዋል? | | | | | | | | | 1. አልተከተብኩም 2. TT-1 ተከትቢያለሁ | | | | | | | | | | | | | | | | | | 1. TT-2 ተከትቢያለሁ | | | | |
| 54 | በዚሀ እርግዝና የደም ማነስ መከላከያ መድሃኒት (አይረን ፎሌት) ወስደዋል? | | | | | | | | | | | | 1. አልወሰድኩም | | | | | | | | | | | | | | | | | 1. ወስጃለሁ | | |
| 55 | በዚህ እርግዝና HIV ምርመራ አድርገዋል? | | | | | | | | | | | | 1. አላደረግኩም | | | | | | | | | | | | | | 1. አድርጊያለሁ | | | | | |
| 56 | በዚህ እርግዝና ወቅት የአንጀት ትላትልን ለማስወገድ መድሃኒት ወስደዋል? | | | | | | | | | | | | 1. አልወሰድኩም | | | | | | | | | | | | | | 1. ወስጃለሁ | | | | | |
| 57 | በዚህ እርግዝና ወቅት የአልጋ አጎበር ተጠቅመዋል? | | | | | | | | | | | | 0. አዎ | | | | | | | | | | | | | | 1. የለም | | | | | |
| 58 | በዚህ እርግዝና ወቅት ህመም አጋጥሞት ነበር? | | | | | | | | | | | | | | | 1. አዎ | | | | | | | | | | | 1. የለም **60** | | | | | |
| 59 | **አዎ** ከሆነ እርግዝና ወቅት ያጋጠመዎት ህመም የትኛው ነበር? | ከባድ የራስ ምታት? | | | | | | | | | | | | | | 1. የለም | | | | | | | | | | | | 1. አዎ | | | | |
|  |  | ከማህፀን ደም ማፍሰስ? | | | | | | | | | | | | | | 1. የለም | | | | | | | | | | | | 1. አዎ | | | | |
|  |  | ማንዘፍዘፍ/ማንቀጥቀጥ? | | | | | | | | | | | | | | 1. የለም | | | | | | | | | | | | 1. አዎ | | | | |
|  |  | የእጅ፤ ፊት ወይም ሙሉ ሰውነት እብጠት? | | | | | | | | | | | | | | 1. የለም | | | | | | | | | | | | 1. አዎ | | | | |
|  |  | ከባድ የራስ ማዞር? | | | | | | | | | | | | | | 1. የለም | | | | | | | | | | | | 1. አዎ | | | | |
|  |  | የሰውነት ትኩሳት/ሙቀት መጨመር? | | | | | | | | | | | | | | 1. የለም | | | | | | | | | | | | 1. አዎ | | | | |
|  |  | የሰውነት ትኩሳትና ብርድ ብርድ ማለት? | | | | | | | | | | | | | | 1. የለም | | | | | | | | | | | | 1. አዎ | | | | |
|  |  | ከማህፀን ደም በብዛት መፍሰስ? | | | | | | | | | | | | | | 1. የለም | | | | | | | | | | | | 1. አዎ | | | | |
|  |  | የደም ግፊት መጨመር? | | | | | | | | | | | | | | 1. የለም | | | | | | | | | | | | 1. አዎ | | | | |
|  |  | እራስን መሳት? | | | | | | | | | | | | | | 1. የለም | | | | | | | | | | | | 1. አዎ | | | | |
|  |  | የሽንት ወይም ሰገራን መቆጣጠር አለመቻል? | | | | | | | | | | | | | | 1. የለም | | | | | | | | | | | | 1. አዎ | | | | |
|  |  | ሽታ ያለው የማህፀን ፈሳሽ? | | | | | | | | | | | | | | 1. የለም | | | | | | | | | | | | 1. አዎ | | | | |
|  |  | የስኳር ህመም | | | | | | | | | | | | | | 1. የለም | | | | | | | | | | | | 1. አዎ | | | | |
| 60 | የእርግዝናው ዓይነት? | | | 1. አንድ | | | | | | 1. ሁለት/መንትያ/ | | | | | | | | | | | | | | | | | | 1. ሶስትና በላይ | | | | |
| 61 | የጨቅላ ህፃኑ ፆታ | | | 1. ወንድ | | | | | | | | | | | | 1. ሴት | | | | | | | | | | | | | | | | |
| 62 | የዚህ እርግዝና ውጤት ምን ሆነ? | | | 1. ጊዜውን ጠብቆ ተወልዷል 2. ሞቶ/ጠፍቶ ተወልዷል 3. እንደተወለደ ሞቷል | | | | | | | | | | | | | | | 1. የፈቃደኝነት ውርጃ 2. የተፈጥሮ ውርጃ | | | | | | | | | | | | | |
| 63 | ልጁ ሲወለድ እንዴት ነበር የመጣው? | | | 1. በጭንቅላቱ 2. በመቀመጫው | | | | 1. በጎን 2. አላውቅም | | | | | | | | | | | | 1. ሌላ ከሆነ ይጠቀስ------------------------ | | | | | | | | | | | | |
| 64 | ህፃኑ ሲወለድ እንዴት ነበር የተወለደው? | | | 1. በማህፀን/በኖርማል 2. በስቲች/ማህፀንን በማስፋት 3. በኢንስትሩሜንታል ዴሊቨሪ/ በመሳሪያ | | | | | | | | | | | | | | | | | 1. በኦፕሬሽን /በሆድ ቀዶ ጥገኛ/ 2. ቆራርጦ መውጣት | | | | | | | | | | | |
| 65 | በጤና ተቋሙ ማን ነበር ያዋለደዎት? | | | | | | | | 1. የህክምና ዶክተር 2. ነርስ | | | | | | | | | | | | | | 1. አዋላጅ ነርስ 2. ጤና መኮንን | | | | | | | | | |
| 66 | በጤና ተቋሙ ያዋለደዎት የጤና ባለሙያ ፆታ ምን ነበር? | | | | | | | | | | | 1. ሴት | | | | | | | | | | | 1. ወንድ | | | | | | | | | |
| 67 | አዋላጇ/ጁ ከማዋለዳቸው በፊት እጃቸውን ታጥበው ነበር? | | | | | | | | | | | 1. አልታጠበችም | | | | | | | | | | | | | | 1. ታጥባለች | | | | | | |
| 68 | አዋላጇ/ጁ የህፃኑን/ኗን የብልት አካባቢ አፅድታለች? | | | | | | | | | | | 1. አላፀዳችም | | | | | | | | | | | | | | 1. አፅድታለች | | | | | | |
| 69 | በወሊድ ወቅት ከአዋለደችዎ/ዎ የጤና ባለሙያ ስለ ህፃናት አደገኛ ምልክቶች የምክር አገልግሎት አግኝተዋል? | | | | | | | | | | | 0. አዎ | | | | | | | | | | | | | | 1. የለም **71** | | | | | | |
| 70 | **አዎ** ከሆነ ስለየትኞቹ የህፃናት አደገኛ ምልክቶች ምክር አገኙ? | | ጡት አለመጥባት | | | | | | | | | 0. አዎ | | | | | | | | | | | | | | 1. የለም | | | | | | |
|  |  |  | ትኩሳት? | | | | | | | | | 0. አዎ | | | | | | | | | | | | | | 1. የለም | | | | | | |
|  |  |  | የሰውነት መቀዝቀዝ | | | | | | | | | 0. አዎ | | | | | | | | | | | | | | 1. የለም | | | | | | |
|  |  |  | መዝለፍለፍ | | | | | | | | | 0. አዎ | | | | | | | | | | | | | | 1. የለም | | | | | | |
|  |  |  | ቶሎ ቶሎ መተንፈስ | | | | | | | | | 0. አዎ | | | | | | | | | | | | | | 1. የለም | | | | | | |
|  |  |  | መንቀጥቀጥ | | | | | | | | | 0. አዎ | | | | | | | | | | | | | | 1. የለም | | | | | | |
|  |  |  | ሆድ መንፋት | | | | | | | | | 0. አዎ | | | | | | | | | | | | | | 1. የለም | | | | | | |
|  |  |  | ደም የቀላቀለ ተቅማጥ | | | | | | | | | 0. አዎ | | | | | | | | | | | | | | 1. የለም | | | | | | |
|  |  |  | ውሃማ ተቅማጥ | | | | | | | | | 0. አዎ | | | | | | | | | | | | | | 1. የለም | | | | | | |
|  |  |  | ሳል | | | | | | | | | 0. አዎ | | | | | | | | | | | | | | 1. የለም | | | | | | |
|  |  |  | የተመገበውን ሁሉ ማስታወክ | | | | | | | | | 0. አዎ | | | | | | | | | | | | | | 1. የለም | | | | | | |
|  |  |  | የእትብት መቅላት፤ መድማት | | | | | | | | | 0. አዎ | | | | | | | | | | | | | | 1. የለም | | | | | | |
|  |  |  | የውስጥ እግሮች፤ እጆች፤ አይን ቢጫ መምሰል | | | | | | | | | 0. አዎ | | | | | | | | | | | | | | 1. የለም | | | | | | |
|  |  |  | መግል የያዘ ሽፍታ | | | | | | | | | 0. አዎ | | | | | | | | | | | | | | 1. የለም | | | | | | |
|  |  |  | የአይን መቅላት | | | | | | | | | 0. አዎ | | | | | | | | | | | | | | 1. የለም | | | | | | |
|  |  |  | የጀሮ ህመም | | | | | | | | | 0. አዎ | | | | | | | | | | | | | | 1. የለም | | | | | | |

**iv. ለጨቅላ ህፃናት የተሰጠ የጤና ክብካቤ ውጤቶች እና በ28ኛው ቀን የጨቅላ ህፃናት ያሉበት ሁኔታ፤**

| **ተ/ቁ** | **ጥያቄ** | | | **ምላሽ /ከፍት ቦታዎችን በመሙላት እና ከአማራጮች ደግሞ አንዱን ይክበቡ** | | | | | | | | | | | | | | | | | | | | | | | | | | |
| --- | --- | --- | --- | --- | --- | --- | --- | --- | --- | --- | --- | --- | --- | --- | --- | --- | --- | --- | --- | --- | --- | --- | --- | --- | --- | --- | --- | --- | --- | --- |
|  |  |  |  | **እንደተወለደ** | | | | **24 ሰዓት** | | | | | **በ 7ኛው ቀን** | | | | | | | **በ14ኛው ቀን** | | | | | | | | | **በ28ኛው ቀን** | |
| 71 | የጨቅላ ህፃን ክብደት? | | | --------ግራም | | | | -------ግራም | | | | | ------ግራም | | | | | | | ------ግራም | | | | | | | | | ---------ግራም | |
| 72 | የጨቅላ ህፃን ቁመት? | | | ---------ሳ.ሜ | | | | --------ሳ.ሜ | | | | | --------ሳ.ሜ | | | | | | | ------ሳ.ሜ | | | | | | | | | ---------ሳ.ሜ | |
| 73 | ህፃኑ የሞት እክል ገጥሞታል? **93** | | | **አዎ** | | | የለም | **አዎ** | | የለም | | | **አዎ** | | የለም | | | | | **አዎ** | | | የለም | | | | | | **አዎ** | የለም |
| 74 | ህፃኑ የህመም እክል ገጠጥሞታል? **93** | | | አዎ | | | **የለም** | አዎ | | **የለም** | | | አዎ | | **የለም** | | | | | አዎ | | | **የለም** | | | | | | አዎ | **የለም** |
| 75 | **አዎ** ከሆነ የትኛው ህመም መቼ አጋጠመው? **(አደገኛ ምልክቶች)** | ጡት አለመጥባት | | አዎ | | | የለም | አዎ | | የለም | | | አዎ | | | የለም | | | | አዎ | | | የለም | | | | | | አዎ | የለም |
|  |  | ትኩሳት? | | አዎ | | | የለም | አዎ | | የለም | | | አዎ | | የለም | | | | | አዎ | | | የለም | | | | | | አዎ | የለም |
|  |  | የሰውነት መቀዝቀዝ | | አዎ | | | የለም | አዎ | | የለም | | | አዎ | | | የለም | | | | አዎ | | | የለም | | | | | | አዎ | የለም |
|  |  | መዝለፍለፍ | | አዎ | | | የለም | አዎ | | የለም | | | አዎ | | | የለም | | | | አዎ | | | የለም | | | | | | አዎ | የለም |
|  |  | ቶሎ ቶሎ መተንፈስ | | አዎ | | | የለም | አዎ | | የለም | | | አዎ | | | የለም | | | | አዎ | | | የለም | | | | | | አዎ | የለም |
|  |  | መንቀጥቀጥ | | አዎ | | | የለም | አዎ | | የለም | | | አዎ | | | የለም | | | | አዎ | | | የለም | | | | | | አዎ | የለም |
|  |  | ሆድ መንፋት | | አዎ | | | የለም | አዎ | | የለም | | | አዎ | | | የለም | | | | አዎ | | | የለም | | | | | | አዎ | የለም |
|  |  | ደም የቀላቀለ ተቅማጥ | | አዎ | | | የለም | አዎ | | የለም | | | አዎ | | | የለም | | | | አዎ | | | | የለም | | | | | አዎ | የለም |
|  |  | ውሃማ ተቅማጥ | | አዎ | | | የለም | አዎ | | የለም | | | አዎ | | | የለም | | | | አዎ | | | | የለም | | | | | አዎ | የለም |
|  |  | ሳል | | አዎ | | | የለም | አዎ | | የለም | | | አዎ | | | የለም | | | | አዎ | | | | የለም | | | | | አዎ | የለም |
|  |  | የተመገበውን ሁሉ ማስታወክ | | አዎ | | | የለም | አዎ | | የለም | | | አዎ | | | የለም | | | | አዎ | | | | የለም | | | | | አዎ | የለም |
|  |  | የእትብት መቅላት፤ መድማት | | አዎ | | | የለም | አዎ | | የለም | | | አዎ | | የለም | | | | | አዎ | | | የለም | | | | | | አዎ | የለም |
|  |  | የውስጥ እግሮች፤ እጆች፤ አይን ቢጫ መምሰል | | አዎ | | | የለም | አዎ | | የለም | | | አዎ | | የለም | | | | | አዎ | | | የለም | | | | | | አዎ | የለም |
|  |  | መግል የያዘ ሽፍታ | | አዎ | | | የለም | አዎ | | የለም | | | አዎ | | የለም | | | | | አዎ | | | የለም | | | | | | አዎ | የለም |
|  |  | የአይን መቅላት | | አዎ | | | የለም | አዎ | | የለም | | | አዎ | | | የለም | | | | አዎ | | | የለም | | | | | | አዎ | የለም |
|  |  | የጀሮ ህመም | | አዎ | | | የለም | አዎ | | የለም | | | አዎ | | | የለም | | | | አዎ | | | የለም | | | | | | አዎ | የለም |
|  |  | ሌላ:------------ | | አዎ | | | የለም | አዎ | | የለም | | | አዎ | | | የለም | | | | አዎ | | | የለም | | | | | | አዎ | የለም |
| 76 | በጤና ተቋም ከወለዱ ከምን ያህል ጊዜ በኋላ ከጤና ድርጅቱ ወደ ቤትዎ ሄዱ? | | | | | | | | | | | | | | | | | -------------ቀናት------------ሰዓታት | | | | | | | | | | | | |
| 77 | ጨቅላ ህፃንዎ ህመሞች ሲያጋጥሙት ህክምና አግኝቷል? | | | | | | | | | | | | | 1. የለም **98** | | | | | | | | | | | 1. አዎ | | | | | |
| 78 | አዎ ካሉ ከምን ያህል ጊዜ በኃላ ህክምና አገኘ? | | | | | | | | | | | | | -----------------ቀናት----------------ሰዓታት | | | | | | | | | | | | | | | | |
| 79 | ለዚህ ህመም እርዳታ ያገኙት ከየት ነው?(ከአንድ በላይ መልስ መስጠት ይቻላል) | | 1. የባህል ህክምና 2. ከመንግስት ሆስፒታል 3. ከጤና ጣቢያ | | | | | | | 1. ጤና ኬላ 2. ከግል ክሊኒክ 3. ከመድሃኒት መደብር/ፋርማሲ | | | | | | | | | | | | | | 1. ከቤት የተዘጋጀ መድሃኒት 2. ሌላ፡--------- | | | | | | |
| 80 | ከህክምና በኃላ የህፃኑ ጤንነት ምን ሁኔታ ላይ ነበር? | | | | | | | | 1. ታክሞ ድኗል | | | | | | | 1. እንደታመመ ነው | | | | | | | | | | | | | 1. ሞቷል | |
| 81 | ለህፃንዎ የድህረ-ወሊድ ህክምና ክትትል አድርገዋል? | | | | | | | | | | | 1. የለም | | | | | | | | | | | | | | 1. አዎ | | | | |
| 82 | ከወለዱ በኃላ በጤና ኤክስቴንሽን ባለሙያ ተጎበኙ? | | | | | | | | | | | 1. አዎ | | | | | | | | | | | | | | 2. የለም **101** | | | | |
| 83 | አዎ ከሆነ ከምን ያህል ጊዜ በኃላ ነበር የተጎበኙት? | | | | | | | | | | | --------------------ቀናት--------------ሳምንታት | | | | | | | | | | | | | | | | | | |
| 84 | ከላይ በተጠቀሱት የጤና ችግሮች ምክንያት እስከ 28 ቀን ዕድሜው ድረስ ቢያንስ ምን ያህል ጊዜ ወደ ህክምና ተቋም ሄደዋል? | | | | | | | | | | | ---------------/ቁጥር/ | | | | | | | | | | | | | | | | | | |
| 85 | በ28ኛው ቀን ህፃኑ በምን ሁኔታ ላይ ነው? | | | | | 1. በጥሩ ሁኔታ በህይወት አለ | | | | | | 1. የአካል ጉዳተኛ ሆኖ በህይወት አለ | | | | | | | | | | | | | | | 1. በህይወት የለም/ሞቷል | | | |
| 86 | ህፃኑ የአካል ጉዳተኛ ከሆነ ጉዳቱ መቼ ተፈጠረ ብለው ያስባሉ? | | | | | | | | | | 1. በተፈጥሮ 2. በወሊድ ወቅት | | | | | | | | 1. ከተወለደ በኋላ 2. አላወቅም | | | | | | | | | | | |
| 87 | የጨቅላ ህፃናት ህመም ሲያጋጥምዎ በቅርበት የሚያገኙት የጤና ተቋም በአቅራቢያዎ አለ? | | | | | | | | | | | | | | | | | | | | | 1. አዎ | | | | | | | 1. የለም | |
| 88 | ከመኖሪያ ቤትዎ ወደ ጤና ተቋሙ በተሸከርካሪ ለመሄድ ምቹ ነው? | | | | | | | | | | | | | | | | | | | | | 0. አዎ | | | | | | | 1. አይደለም | |
| 89 | የመኖሪያ ቤትዎ በአቅራቢያው ካለው የጤና ጣቢያ በምን ያህል ይርቃል ወይም ምን ያህል ጊዜ ይወስዳል?*(መላሽዋ መገመት ካልቻሉ መልሱን ጠያቂው በመገመት ይሙሉት)* | | | | | | | | | | | | | | | | | | | | | ----------------ኪሎ ሜትር  ---------ደቂቃ--------ሰዓት | | | | | | | | |
| 90 | ወደ ጤና ጣቢያ ለመሄድ ምን ዓይነት መጓጓዣ ይጠቀማሉ? *(ከአንድ በላይ መልስ ከመለሱ ያክብቡት)* | | | | | | | | 1. በእግር 2. በባህላዊ ሸክም ወይም ስትሬቸር 3. በፈረስ ወይም በቅሎ | | | | | | | | | | | | | | | | | | | 1. በጋሪ 2. በተሸከርካሪ | | |
| 91 | ወደ ጤና ጣቢያ ለመሄድ ምን ዓይነት መንገድ ይጠቀማሉ? | | | | 1. መንገድ የለም 2. በሁሉም የአየር ንብረት መሄድ የሚያስችል መንገድ | | | | | | | | | | | | | 1. በበጋ ብቻ የሚያስኬድ መንገድ 2. አስፋልት መንገድ | | | | | | | | | | | | |
| 92 | በአካባቢዎ ባለው ትራንስፖርት ዓይነት ተጠቅመው ከመኖሪያ ቤትዎ ወደ ጤና ጣቢያ ቢሄዱ በአማካኝ ምን ያህል ጊዜ ይፈጃል? *(መላሽዋ መገመት ካልቻሉ መልሱን ጠያቂው በመገመት ይሙሉት)* | | | | | | | | | | | | | | | | | | | | ----------ደቂቃ-------ሰዓት | | | | | | | | | |
| 93 | ቤትዎ ከጤና ኬላው ምን ያህል ይርቃል? *(መላሽዋ መገመት ካልቻሉ መልሱን ጠያቂው በመገመት ይሙሉት)* | | | | | | | | | | | | | | | | | | | | ----------------ኪሎ ሜትር | | | | | | | | | |
| 94 | በአካባቢዎ ባለው ትራንስፖርት ዓይነት ተጠቅመው ወደ ጤና ኬላ ቢሄዱ በአማካኝ ምን ያህል ጊዜ ይፈጃል? *(መላሽዋ መገመት ካልቻሉ መልሱን ጠያቂው በመገመት ይሙሉት)* | | | | | | | | | | | | | | | | | | | | ----------ደቂቃ-------ሰዓት | | | | | | | | | |
| 95 | በአጠቃላይ የጨቅላ ህፃናት ህክምና ለማግኘት አስቸጋሪ ነው ብለው ያምናሉ? | | | | | | | | | | | | | | | | 1. አዎ | | | | | | | | | | | 1. አላምንም | | |

***መረጃውን በመስጠት ስለተባበሩን እናመሰግናለን!!!***
