# Supplementary material for: Prevalence of common symptoms of neonatal illness in Northwest Ethiopia: A repeated measure cross-sectional study
Source: PLoS One. 2021 Mar 30;16(3):e0248678. doi: 10.1371/journal.pone.0248678 (PMC8009397; doi:10.1371/journal.pone.0248678)
Supplement: S4 Annex — (DOCX) [file pone.0248678.s004.docx]

**Annex 4:** Obstetric characteristics of mothers and delivery assistants in selected health facilities, northwest Ethiopia (N=338), March 2019.

| **Variable** | **Category** | **N** | **%** |
| --- | --- | --- | --- |
| 1. **Pregnancy and delivery characteristics** | | | |
| Age at first pregnancy (years) | Before 18 | 66 | 19.5 |
|  | ≥ 18 | 272 | 80.5 |
| Gestation age (weeks) | Preterm /≤36/ | 48 | 14.2 |
|  | Term /37-41/ | 284 | 84.0 |
|  | Post-term /≥42/ | 06 | 1.8 |
| Lifetime pregnancy (number) | 1 – 3 times | 236 | 69.8 |
|  | ≥ 4 times | 102 | 30.2 |
| Number of live children | 0 – 3 children | 256 | 75.7 |
|  | ≥ 4 children | 82 | 24.3 |
| Experience of neonatal death | None | 311 | 92 |
|  | 1 – 3 times | 27 | 8 |
| ANC at current pregnancy | No | 53 | 15.7 |
|  | Yes | 285 | 84.3 |
| Health education by HEW | No | 201 | 59.5 |
|  | Yes | 137 | 40.5 |
| Current pregnancy | Inappropriate time | 45 | 13.3 |
|  | Wanted/planned | 293 | 86.7 |
| Birth control use before the current pregnancy | No | 94 | 27.8 |
|  | Yes | 244 | 72.2 |
| Frequency of Tetanus antitoxin intake (number) | 0 | 91 | 26.9 |
|  | 1 | 46 | 13.6 |
|  | 2 | 201 | 59.5 |
| Type of pregnancy | Single | 323 | 95.6 |
|  | Twin or triplet | 15 | 4.4 |
| Type of delivery | SVD | 285 (84.3) | 84.3 |
|  | Instrumental (CS, etc.) | 53 (15.7) | 15.7 |
| 1. **Skilled birth attendant characteristics** | | | |
| Profession | Medical Doctor | 10 | 3 |
|  | Nurse | 14 | 4.1 |
|  | Midwife | 295 | 87.3 |
|  | Health Officer | 19 | 5.6 |
| Sex of the delivery assistant | Female | 151 | 44.7 |
|  | Male | 187 | 55.3 |
| Delivery assistant washed hands before assisting the delivery | No | 150 | 44.4 |
|  | Yes | 188 | 55.6 |
| Delivery assistant cleaned the perineum of the newborn | No | 37 | 10.9 |
|  | Yes | 301 | 89.1 |
| Delivery assistant counseled on neonatal danger signs | No | 159 | 47 |
|  | Yes | 179 | 53 |
