# Supplementary material for: Prevalence of common symptoms of neonatal illness in Northwest Ethiopia: A repeated measure cross-sectional study
Source: PLoS One. 2021 Mar 30;16(3):e0248678. doi: 10.1371/journal.pone.0248678 (PMC8009397; doi:10.1371/journal.pone.0248678)
Supplement: S5 Annex — (DOCX) [file pone.0248678.s005.docx]

**Annex 5:** Distribution of neonatal illnesses with birth dates among health facility deliveries in northwest Ethiopia, March 2019.
